# Recent developments and applications of selected ion flow tube mass spectrometry (SIFT‐MS)

**DOI:** 10.1002/mas.21835

**Published:** 2023-02-12

**Authors:** David Smith, Patrik Španěl, Nicholas Demarais, Vaughan S. Langford, Murray J. McEwan

**Affiliations:** ^1^ J. Heyrovský Institute of Physical Chemistry, Czech Academy of Sciences Prague Czechia; ^2^ Syft Technologies Limited Christchurch New Zealand; ^3^ Department of Chemistry University of Canterbury Christchurch New Zealand

**Keywords:** cation and anion gas phase chemistry, nitrogen carrier gas, selected ion flow tube mass spectrometry, SIFT‐MS, VOCs, volatile organic compounds

## Abstract

Selected ion flow tube mass spectrometry (SIFT‐MS) is now recognized as the most versatile analytical technique for the identification and quantification of trace gases down to the parts‐per‐trillion by volume, pptv, range. This statement is supported by the wide reach of its applications, from real‐time analysis, obviating sample collection of very humid exhaled breath, to its adoption in industrial scenarios for air quality monitoring. This review touches on the recent extensions to the underpinning ion chemistry kinetics library and the alternative challenge of using nitrogen carrier gas instead of helium. The addition of reagent anions in the *Voice200* series of SIFT‐MS instruments has enhanced the analytical capability, thus allowing analyses of volatile trace compounds in humid air that cannot be analyzed using reagent cations alone, as clarified by outlining the anion chemistry involved. Case studies are reviewed of breath analysis and bacterial culture volatile organic compound (VOC), emissions, environmental applications such as air, water, and soil analysis, workplace safety such as transport container fumigants, airborne contamination in semiconductor fabrication, food flavor and spoilage, drugs contamination and VOC emissions from packaging to demonstrate the stated qualities and uniqueness of the new generation SIFT‐MS instrumentation. Finally, some advancements that can be made to improve the analytical capability and reach of SIFT‐MS are mentioned.

## 1 INTRODUCTION

Veronica Bierbaum adopted the selected ion flow tube (SIFT) shortly after its inception in 1976. Together with her mentor, Charles DePuy, she was the first to exploit its use for the study of gas phase negative ion–molecule reactions and is thus rightly considered to be one of the SIFT pioneers (DePuy et al., 1980, 1982). Her scientific acumen is matched by her giving, charming personality, which has impressed the worldwide chemistry community. Veronica has promoted the work on ion–molecule reactions with great imagination and energy, as befits such an exceptional scientist, and she has expertly trained many doctoral students and postdoctoral researchers in this topic, including one of the authors of this review, Nicholas Demarais, and the guest editors of this special issue. It is fitting that a special issue of Mass Spectrometry Reviews is dedicated to Veronica, and we are proud and pleased to be contributors.

Selected ion flow tube‐mass spectrometry (SIFT‐MS) has now become a widely used analytical instrument in diverse fields of science and technology. It has its origins in the SIFT technique, which was conceived by Adams and Smith (1976) for the measurement of the kinetics of ion–molecule reactions occurring in the terrestrial ionosphere and interstellar gas clouds (Smith & Španěl, 1995). Following the demonstrations of its great versatility in the study of gas‐phase ionic reactions (Adams et al., 1980; Smith et al., 1978), it was quickly adopted in other laboratories worldwide, among the first being that of DePuy et al. (1980, 1982), to whom this review article is dedicated.

In 1996, some 20 years after its inception, Smith and Španěl (1996a, 1996b) and Španěl and Smith (1996) described the details of SIFT and the contribution it can make to online analyses of volatile organic compounds (VOCs) in ambient air, including very humid air such as exhaled breath. Focused research and development over several years have realized SIFT‐MS as a viable and practical analytical technique (Španěl & Smith, 1999). Initial developments were carried out using very large, laboratory‐based SIFT‐MS instruments. These have evolved into much smaller and readily transportable instruments that have been commercialized and are being exploited for basic research, including breath analysis, environmental air analysis, and food science, and in the industrial setting for real‐time air monitoring in the semiconductor industry and the air in containers at ports to detect illicit materials and to protect the health and safety of customs officials.

The first detailed review of SIFT‐MS was published in *Mass Spectrometry Reviews, MSR* (Smith & Španěl, 2005), covering the basic principles of this technique, an overview of the underlying ion chemistry and illustrative examples of quantification of trace VOCs, including analyses of breath, the headspace of biological fluids and exhaust gases from combustion engines. The next comprehensive review, also in *MSR*, appeared in 2011 (Španěl & Smith, 2011) covering the finer important details of SIFT‐MS, such as the need to account for differential ionic diffusion and mass discrimination for accurate quantification. Examples of the progress made included the characterization of reference ranges for common breath metabolites and the assessment of the contribution of orally‐generated compounds to the composition of exhaled breath. The historical perspective and development of SIFT‐MS from SIFT and the similar flowing afterglow are given in detail in these two *MSR* reviews and need not be repeated in the present review.

However, it is important to mention two types of small instruments used for SIFT‐MS research in the last 12 years: the *Profile 3* developed in the United Kingdom (Instrument Science Limited) and the *Voice200* developed in New Zealand (Syft Technologies Limited). Just a few *Profile 3* have been produced that have been used to investigate the ion chemistry of H_3_O^+^, NO^+^, and O_2_^+•^ reagent cations with several classes of VOCs to construct the kinetics library for SIFT‐MS quantification, and as research instruments for exhaled breath and biological fluid headspace analysis, as referred to later in this review. The two MSR papers referenced above largely describe the *Profile 3* instruments and the research carried out by their exploitation. This generation of small SIFT‐MS instruments has a very short flow tube (~5 cm) and operates at room temperature carrier gas (Smith et al., 2009).

The *Voice200* class of SIFT‐MS instruments were developed at a much higher engineering standard and have been produced in much larger numbers. It is successful worldwide in many industrial, trade, and research areas where trace gas analysis is needed (Dummer et al., 2013; Francis et al., 2009). They also have a longer flow tube (~17 cm) that can be heated up to 140°C to benefit the reagent ion formation, as described later. Some of the *Voice200* instruments have the precious facility to enable switching between cation and anion reagents (i.e., dual‐polarity) and thus can analyze trace compounds that cannot be achieved with just reagent cations.

Several characteristics of SIFT‐MS have made it an attractive option for routine analytical and testing laboratories: direct analysis of volatiles with minimal or no sample preparation, flexible sample delivery (headspace, sample bags, canisters, thermal desorption tubes), high sensitivity, high specificity, single analysis of a wide range of compounds, and high repeatability. Typical considerations for the adoption of a new technique for routine testing include:

(1) automation (for reduced staff cost and increased precision),
(2) compatibility with conventional sample preparation procedures,
(3) ability to validate analytical methods,
(4) compatibility with quality control protocols (including regular calibration), and
(5) comparable results with techniques based on the relevant “gold standard” analysis.

Recent publications have demonstrated the suitability of SIFT‐MS for routine headspace analysis across all these areas (Hastie et al., 2021; Perkins & Langford, 2021a, 2021b).

During the 10 years since the publication of the 2011 *MSR* article, much progress has been made in optimizing the *Profile 3* and *Voice200* instruments in understanding the complex analytical ion chemistry involved and their wider applications in research and industry. It is now appropriate to review this progress covering the following topics. The underlying H_3_O^+^, NO^+^, and O_2_^+•^ cation chemistry is reprised, including the major developments that have broadened the scope and applications of SIFT‐MS to include the adoption of nitrogen as the carrier gas (principally because of supply and cost considerations). The investigation of the more complex ion chemistry involved and widening the range of compounds that can be analyzed by exploiting reagent anions such as O^−^ and OH^−^ for analysis are also discussed. Breath and headspace of biogenic fluids, environmental monitoring and applications, workplace safety and indoor air quality, food VOCs, and pharmaceutical and materials emissions analysis are described as examples of the widening applications of SIFT‐MS, which has become a mainstream analytical tool for analysis. A final section includes summary remarks on its successes and the ongoing developments and potential future applications of SIFT‐MS.

## 2 ION CHEMISTRY BACKGROUND OF SIFT‐MS

The details of the basic principles of SIFT‐MS have been well established for about a quarter of a century. They have been well explained by several review and research papers (Smith & Španěl, 2011; Španěl & Smith, 2011, 2013; Španěl et al., 2006) and need not be included in this review, but a brief reprise of the cation ion chemistry (and later the anion chemistry) is appropriate since it will facilitate an understanding of the new developments to be discussed in this review. In short, soft chemical ionization of trace gases, VOCs, in air is exploited using selected reagent ions reacting with the trace compounds, usually VOCs, present in an air sample introduced into a fast flow tube reactor at a known rate. The reagent and analyte ions are detected downstream using a quadrupole mass spectrometer (QMS). This provides the data needed to calculate the concentrations of each identified neutral analyte in the sample. Reagent cations (and anions) can be formed in a microwave discharge through moist air at a pressure of typically 0.5 mbar. After selection by *m/z* in a quadrupole mass filter, they are injected into the carrier gas at laboratory energies sufficiently low that collisions with the carrier gas atoms or molecules do not dissociate them. Until recently, the fast‐flowing carrier gas was always helium at a pressure of about 1 mbar. However, nitrogen is now being used as the carrier gas at a lower pressure of about 0.4 mbar, which creates some new challenges, as we discuss in a later subsection 3.2. Data can be collected using either the full scan (FS) mode of the analytical QMS as a regular mass spectrum or the multiple ion monitoring (MIM) mode (sometimes termed the selected ion mode [SIM]), in which the QMS is rapidly switched between chosen *m/z* values of the reagent and analyte ions, which are counted over chosen *m/z* dwell periods by the post‐QMS detection system and analyzed for the VOC concentrations.

Identification and correct quantification of the neutral trace VOCs demands a proper understanding of the rate coefficients, the product ions of the analytical ion‐molecule reactions and of related physical parameters, such as the carrier gas and sample gas flow rates. The choice of the reagent ion for analysis of a particular VOC is predicated on the nature of the VOC. H_3_O^+^ is the most flexible reagent, with NO^+^ also very valuable. Rapid switching between these reagent ions is a very important facility. The measurements can be performed rapidly such that time‐varying concentrations can be determined, for example, of the metabolites in single breath exhalations. The details of the calculation of concentrations of analytes from the raw SIFT‐MS data in the form of ion count rates have been discussed previously (Španěl & Smith, 2013). This procedure inherently presumes the correct identification of analytes and their assignment to characteristic product ions. Any overlaps between the analytes in the mixture will complicate this and require careful development of the quantification methods.

A great deal of effort has been given to the construction of a library of rate coefficients and product ions that can be in‐built into the SIFT‐MS instruments and provide simultaneous analyses of the several VOCs present in a sample. This has required SIFT measurements of hundreds of reactions of the reagent cations H_3_O^+^, NO^+^, and O_2_^+•^ with many classes of VOCs such as alcohols, aldehydes, ketones, and so forth, with (variously) 10–20 compounds included for each class of the VOC. While significant works have been conducted by Bierbaum and others to expand the negative ion library (Bierbaum, 2015; Custer et al., 2003; DePuy & Bierbaum, 1987), fewer reactions of the reagent anions O^−^, OH^−^, NO_2_^−^, and so forth, have been studied, but the number continues to grow. This is rather demanding work, akin to the building of an extensive library of gas chromatography–MS (GC‐MS) spectra, but just as necessary. This is an ongoing effort as more compounds fall into the scope of SIFT‐MS analysis, and kinetics data are required. These measurements have revealed much about the principles of ion chemistry under the thermalized conditions that exist in SIFT‐MS flow tube reactors. The general features of the analytical reagent cation reactions are now discussed to facilitate understanding of the applications of SIFT‐MS that are discussed in later sections.

### 2.1 H_3_O^+^, NO^+^, O_2_^+•^ reagent cation reactions with VOCs; summary features

(i) **H**_**3**_ **O** ^**+**^
  The hydronium cation is the most widely exploited reagent ion in SIFT‐MS. It will transfer its proton exothermically to VOCs that have proton affinities (PA) greater than that of the water molecule, which is a great fraction of VOCs. We refer to this simple principle later when considering the isoelectronic ammonium cation, NH_4_^+^, as a potential reagent cation. The basic premise is that exothermic proton transfer, PT, can occur on every collision of the reactant molecule, M, as long as the exothermicity of the proton transfer reaction exceeds about 25 kJ/mol (Bouchoux et al., 1996):
(1) $$H_{3}O^{+}+M\to {\text{MH}}^{+}+H_{2}O.$$
  Then, the rate coefficient for the reaction, *k*, is equal to the collisional rate coefficient, *k*_*c*_, which can be calculated (Su & Chesnavich, 1982). Note that this is only the case for exothermic PT and does not apply to other types of reactions. This assumption is commonly adopted in SIFT‐MS analysis (Španěl & Smith, 2013). MH^+^ can be the analyte ion for the analysis of M if it is stable and detected by the analytical MS. However, fragmentation of the nascent (MH^+^)* can occur in some PT reactions. This is a very common occurrence for the reaction of H_3_O^+^ with aliphatic alcohols, even the simpler ones such as 1‐propanol:
(2a) $$H_{3}O^{+}+C_{3}H_{7}\text{OH}\to C_{3}H_{7}^{+}+2H_{2}O,$$
(2b) $$\to \;C_{3}H_{7}{\text{OH}}_{2}^{+}+H_{2}O.$$
  For the longer chain alcohols, such fragmentation is dominant (Španěl & Smith, 1997). There are many more classes of VOCs for which this phenomenon occurs, including reactions of H_3_O^+^ with some aldehydes, carboxylic acids, and esters (Smith & Španěl, 2005). Fragmentation in PT from H_3_O^+^ may be considered the rule rather than the exception, but it is not always predictable. The omission of fragment analyte ions and the tendency to adopt only the parent MH^+^ ions for analysis are common mistakes that lead to erroneous quantification. The general reaction behavior of each class of compounds on protonation by H_3_O^+^ is tabulated in several review papers (Smith & Španěl, 2005, 2011).
  A phenomenon that impacts all SIFT‐MS analyses is the appearance of hydrates of both the reagent ions and the analyte ions, especially when analyzing humid mixtures such as exhaled breath. Most obvious is the production of the H_3_O^+^(H_2_O)_1,2,3_ hydrates that are mostly formed in termolecular reactions of H_3_O^+^ with H_2_O molecules. As we will show later, these hydrates become more obvious when using nitrogen rather than helium as the carrier gas. Also, when protonated stable molecules MH^+^ are produced, they also can become efficiently hydrated by both association reactions with H_2_O molecules and by ligand switching reactions of H_2_O molecules in the H_3_O^+^ hydrates by trace VOC molecules, M:
(3) $$H_{3}O^{+}\left(H_{2}O\right)+M\to {\text{MH}}^{+}H_{2}O+H_{2}O.$$
  For some classes of VOCs, only the monohydrates MH^+^H_2_O are formed; for other classes, the dihydrates MH^+^(H_2_O)_2_ and trihydrates MH^+^(H_2_O) are also formed (Smith et al., 2014, 2019; Španěl et al., 2017b). With increasing sample humidity, dihydrate and trihydrate ions increase and can become the dominant analyte ions. These hydrates must be included as analyte ions for accurate analyses to be realized (Španěl & Smith, 2013; Španěl et al., 2006). A most valuable aspect of the formation of hydrated hydronium ions in SIFT‐MS is that they provide the means to measure the absolute humidity of a sample, which is important in many studies (Španěl & Smith, 2001). An interesting and important point to be made is that heating the carrier gas can inhibit the formation of hydrates, as effected in the *Voice200* series of instruments (Prince et al., 2010).
(ii) **NO** ^**+**^
  A great feature of SIFT‐MS is the availability of the different reagent cations and anions and the facility to rapidly switch between them. This is especially analytically helpful because of the very different processes involved in their reactions with different classes of VOCs. The differences in the gas‐phase reactions of this nitrosonium cation NO^+^ compared to H_3_O^+^ are a real boon for analysis. Whereas H_3_O^+^ transfers a proton to most (but not all) VOCs, the reactions of NO^+^ are very different and diverse. The closed‐shell NO^+^ ground‐state ion has a recombination energy of 9.26 eV, so it can charge exchange with VOC molecules that have an ionization energy IE(M) less than 9.26 eV. This encompasses a significant number of VOCs and so charge exchange can be exploited for analysis using NO^+^ reagent ions, such as for toluene:
(4) $${\text{NO}}^{+}+C_{6}H_{5}{\text{CH}}_{3}\to C_{7}H_{8}^{+•}+{\text{NO}}^{•}.$$
  Although this is a “soft ionization” process, it sometimes results in partial fragmentation of the nascent (M^+^)* ion. The low IE of most amines, alkenes, and terpenes allow charge transfer to proceed in their reactions with NO^+^ (Smith & Španěl, 2005).
  Other recognizable processes are involved in the reactions of NO^+^ with specific VOCs, which often result in only one or two product ions. These are hydride ion (H^−^) transfer producing (M−H)^+^ ions, such as the ethanol reaction:
(5) $${\text{NO}}^{+}+C_{2}H_{5}\text{OH}\to C_{2}H_{5}O^{+}+\text{HNO}.$$
  Hydroxide ion (OH^−^) transfer producing (M–OH)^+^ ions. This often occurs in the reactions with branched‐chain alcohols such as 2‐methyl‐2‐propanol:
(6) $${\text{NO}}^{+}+{\left({\text{CH}}_{3}\right)}_{3}\text{COH}\to C_{4}{H_{9}}^{+}+{\text{HNO}}_{2}.$$
  Alkoxide ion (OR^−^) transfer producing (M−OR)^+^ ions often occurs in parallel with adduct ion formation that produces NO^+^M ions. This branching is very common for reactions of NO^+^ with esters, as for the methyl acetate reaction:
(7a) $${\text{NO}}^{+}+{\text{CH}}_{3}{\text{COOCH}}_{3}+\text{He}\to {\text{NO}}^{+}{\text{CH}}_{3}{\text{COOCH}}_{3}+\text{He},$$
(7b) $$\to {\text{CH}}_{3}{\text{CO}}^{+}+{\text{CH}}_{3}{\text{NO}}_{2}.$$
  These various reaction processes and their connection to different VOCs are tabulated in previous publications (Smith & Španěl, 2011; Španěl & Smith, 2011). Understanding the connection of these NO^+^ reaction processes to particular VOCs can be very helpful in trace compound identification. Like most exothermic proton transfer reactions, most of these varied NO^+^ reactions proceed at or close to their respective collisional rates (*k* = *k*_*c*_), which greatly facilitates SIFT‐MS analyses of the respective VOCs. The reaction channel (7a) is an association reaction producing the adduct ion, which is a very important process in SIFT‐MS. Such reactions can be termolecular when the rate will be dependent on the temperature, pressure, and nature (helium or nitrogen) of the bath (carrier) gas. In this process, the excited nascent adduct ion (NO^+^M)* is stabilized against spontaneous dissociation back to the reactants by atomic or molecular super‐elastic collisions (He or N_2_). Alternatively, the association may be bimolecular when the bond energy is distributed between the vibrational modes of the excited nascent adduct ion, which is thus stabilized against dissociation. This bimolecular association is a recent realization that we discuss later in this review. Adduct ion formation is a common process in the reactions of NO^+^ with several types of VOCs, including alkenes (Michalcikova et al., 2018), aldehydes (Smith et al., 2014), alcohols (Španěl et al., 2017b), ketones (Spesyvyi et al., 2017), and carboxylic acids (Michalčíková & Španěl, 2014). It is especially valuable for the analyses of monoketones (Smith et al., 2019) when the analyte ion can be recognized as 30 *m/z* units above the molecular mass of the reactant neutral VOC.
  It should be mentioned that NO^+^ ions react with H_2_O molecules by termolecular association producing NO^+^(H_2_O)_1,2_ adduct ions. This monohydrate ion is usually a few percent of the precursor NO^+^ and so it is routinely included as a reagent ion in SIFT‐MS analyses. It can be reactive with some VOCs by ligand switching producing NO^+^M adduct ions, and this must be considered when interpreting the SIFT‐MS spectra (Smith et al., 2003).
(iii) **O**_**2**_^**+•**^

The high recombination energy of the open‐shell ground state dioxygenyl O_2_^+•^ radical cation, 12.07 eV, relative to NO^+^ (9.26 eV), allows charge transfer to occur to some compounds that do not react with H_3_O^+^ and NO^+^ so that it can be a valuable reagent ion for SIFT‐MS. But this charge transfer process almost always results in partial fragmentation of the nascent parent VOC cation. Thus, the analyses of samples with many VOCs can result in very complex spectra with many *m*/*z* overlaps, which can be futile, especially when the common open‐shell polyatomic ion products usually hydrate (Smith et al., 2019). Because of these unsurmountable problems, O_2_^+•^ reagent ions are most valuable for detecting and quantifying small molecules for which the product parent cations do not dissociate. Examples are NH_3_, NO, NO_2_ (volatile inorganic compounds, not VOCs), and CS_2_, relevant species in environmental monitoring.

(8) $$O_{2}^{+•}+({\text{NH}}_{3},{\text{NO}}^{•},{\text{NO}}_{2}^{•},{\text{CS}}_{2})\;\to ({\text{NH}}_{3}^{+},{\text{NO}}^{+},{\text{NO}}_{2}^{+},{\text{CS}}_{2}^{+•})+O_{2}.$$

Small open‐shell cations are produced in these reactions. O_2_^+•^ ions react with water molecules only by slow termolecular association producing O_2_^+•^(H_2_O)_1,2_ hydrates, but these do not significantly complicate SIFT‐MS analyses. As we show later in this review, O_2_^+•^ reagent ions can be used in SIFT‐MS to analyze the biologically important polyatomic n‐pentane (C_5_H_12_) and methane (CH_4_), which do not react with H_3_O^+^ and NO^+^ in SIFT‐MS.

A very thorough review of the ion chemistries of NO^+^ and O_2_^+•^ for the analyses of various classes of compounds by SIFT‐MS and PTR + SRI‐MS has recently been published in *MSR* (Hegen et al., 2022).

### 2.2 Reagent anions

The use of reagent anions in SIFT‐MS has been pioneered by Syft Technologies in Christchurch, New Zealand. They have developed a remarkably versatile *Voice200* commercial instrument that in its dual‐polarity variant utilizes both cations and anions for gas‐phase analysis (Hera et al., 2017). Because this is a relatively recent innovation, much less work has been published in the scientific literature on the use of reagent anions in SIFT‐MS. The *Profile 3* instruments do not have the anion reagent ion facility. Like reagent cations, viable reagent anions must not react rapidly with the major components of air or the carrier gas. Thus, the possible reagent anions are seen to be O^−•^, OH^−^, O_2_^−•^, NO_2_^−^, and NO_3_^−^. Fortunately, they are stable and unreactive with N_2_ carrier gas molecules and can also be generated in a microwave discharge through moist air. The humidity needs to be adjusted to optimize particular reagent anions. O^−•^, NO_2_^−^, and NO_3_^−^ are obtained from dry air and OH^−^ and O_2_^−•^ from moist air. The range of analytes open to the SIFT‐MS technique has been extended and selectivity enhanced using negative reagent ions to include a range of halogenated compounds and sulphur oxides.

## 3 RECENT ION CHEMISTRY STUDIES EXTENDING THE SIFT‐MS KINETICS LIBRARY

This section summarizes the studies of the kinetics of ion–molecule reactions related to SIFT‐MS carried out since 2011. The readers who are more interested in the recent SIFT‐MS applications are advised to skip straight to Section 4.

### 3.1 Reagent cations

As mentioned previously, the extension of the SIFT‐MS kinetics library is an ongoing process. Further additions are stimulated by research work when new compounds are discovered in real samples, such as exhaled breath and cell culture headspace, which necessitate kinetics data for their analysis. Thus, SIFT measurement of H_3_O^+^, NO^+^, and O_2_^+•^ reagent ions with more classes of VOCs and groups of isomeric and isobaric VOCs are carried out, principally using *Profile 3* instruments, not only to provide the necessary rate coefficients and product ions but also to gain insight into the reaction processes involved. Many such studies have been conducted since the last review of the SIFT‐MS‐related kinetics (Smith & Španěl, 2005). They include studies of the reactions of six volatile phytogenic esters (Sovová et al., 2011) and biogenic isobaric compounds at molecular weight (MW) of 86 u: 2,3‐butanedione (diacetyl), C_4_H_6_O_2_; allyl ethyl ether, C_5_H_10_O cyclopropane carboxylic acid, C_4_H_6_O_2_: ‐butyrolactone, C_4_H_6_O_2_. At MW 88 u: 3‐hydroxybutanone (acetoin), C_4_H_8_O_2_; n‐butyric acid, C_4_H_8_O_2_; ethyl acetate, C_4_H_8_O_2_; pyruvic acid, C_3_H_4_O_3_ (Smith et al., 2011), which are relevant to plant and human biology. Also, the reactions of seven isomers of hexanol (Smith et al., 2012) that are commonly met in food sciences were studied. Such kinetics data and those for volatile aldehydes, fatty acids, and some sulfides are all utilized for the analysis of volatiles emitted by processed foodstuffs, well exemplified by emissions from lipid oxidation of beef (Olivares et al., 2012). Aldehydes are released by many biogenic systems and are a continuous focus of SIFT‐MS research; hence the need for kinetics data (e.g., Smith et al., 2014). This is also true for alcohols and ketones; hence the detailed studies of the reactions of a series of primary alcohols (Španěl, Zabka, et al., 2017) and of the reactions of several monoketones and diones that also focused on the hydration of the product ions of these reactions (Smith et al., 2019).

In addition to these surveys involving numbers of VOCs in different classes, several shorter studies of specific VOCs have been carried out that required kinetics data. Of note is the approach to measuring n‐pentane (C_5_H_12_) by SIFT‐MS (Dryahina et al., 2013). This hydrocarbon does not react with either H_3_O^+^ or NO^+^, but it does react by dissociative charge transfer with O_2_^+•^ producing four product ions. To avoid analyte ion overlaps, only two of the product ions, C_3_H_6_^+•^ and C_5_H_12_^+•^ could be used to quantify n‐pentane and so accurate measurements of the rate coefficient and product ion branching ratio of the overall reaction and how the branching ratios were influenced by water vapor were needed. The results of this detailed study allowed pentane to be measured in humid exhaled breath, which will be discussed in the later section of this paper on breath analysis.

Further to this, a detailed study was carried out to obtain the kinetics data for quantifying malondialdehyde (MDA) in biogenic fluids by measuring the rate coefficient and product ions for its reactions with H_3_O^+^, NO^+^, and O_2_^+•^ (Shestivska, Antonowicz, et al., 2015). This provided the means for SIFT‐MS analysis of MDA in humid air and the headspace of liquid‐phase biogenic media and, perhaps, exhaled breath. Since MDA reflects free oxygen‐radical lipid peroxidation, it can be useful as a biomarker to track this process.

A recent ion chemistry study (Smith & Španěl, 2022), which could impact many SIFT‐MS analytical studies of different classes of VOC, involves the reactions in the helium carrier gas of H_3_O^+^, NO^+^, and O_2_^+^, with the major gases in air and breath samples, viz. N_2_, O_2_, CO_2_, and H_2_O that are usually considered to be inert. These reactions form weakly‐bound adduct ions by slow ternary association reactions ranging in *m/z* from 47 (H_3_O^+^N_2_) to 76 (O_2_^+•^CO_2_). While the formation rate of these “ghost peaks” is relatively slow, they can reach peak heights in SIFT‐MS spectra comparable to and sometimes greater than the analyte peaks due to reagent ion reactions with the analyte VOC present at low concentrations. These “ghost peaks” must not be mistaken for genuine VOC analyte ions. It is likely that the signals of these “ghost peaks” are likely to be greater when using nitrogen carrier gas, which is more efficient in stabilizing nascent excited adduct ions against spontaneous dissociation to the reactants.

The availability of the extensive kinetics library of the reaction of these reagent cations has allowed analytical data to be obtained in many areas of research, not least in breath and biogenic liquids headspace that we summarize in the next section of this review. A brief summary of some of the other areas investigated during the last 10 years, principally using *Profile 3* instruments, is now given here.

Somewhat outside our general interest in trace gas analysis but no less interesting are analyses of the products of laser ablation of FOX‐7 explosive (Civiš et al., 2011) and the decomposition products of other explosives and ballistic products (Civis et al., 2016). Further, various ion–molecule reactions occurring in planetary atmospheres have been reported (Zymak et al., 2016, 2020). The release of ammonia from heated cannabis and its relation to THC content is of great interest since the toxic ammonia is known to result in neurobehavioral impairment (Smith et al., 2015). The volatile compounds generated by the electrochemical reduction of atmospheric carbon dioxide and nitrogen include isobaric methanol (CH_3_OH) and hydrazine (N_2_H_4_), and a careful study has allowed these two isobaric compounds to be quantified by SIFT‐MS when coexisting in a humid air mixture (Shestivska et al., 2020). Similarly, isobaric analyte ion overlap can occur when analyzing biogenic media for coexisting acetaldehyde, dimethyl sulfide, and carbon dioxide, and a study has indicated how this can be resolved (Smith et al., 2013).

During the past few years, interest has grown in detecting monoterpenes released into the atmosphere and present in exhaled breath. Thus, several analytical studies of several isomers of monoterpenes (general molecular formula C_10_H_16_) have been carried out to investigate if the different isomers could be separately identified and quantified by SIFT‐MS in combination with GC, using either collisional dissociation, thermal desorption, or unorthodox reagent ions (Lacko et al., 2019; Sovova et al., 2019; Spesyvyi et al., 2016, 2019). As an additional variable, the influence of bath gas (helium, nitrogen) on monoterpene analysis was recently studied (Španěl et al., 2022).

**Figure 1:**
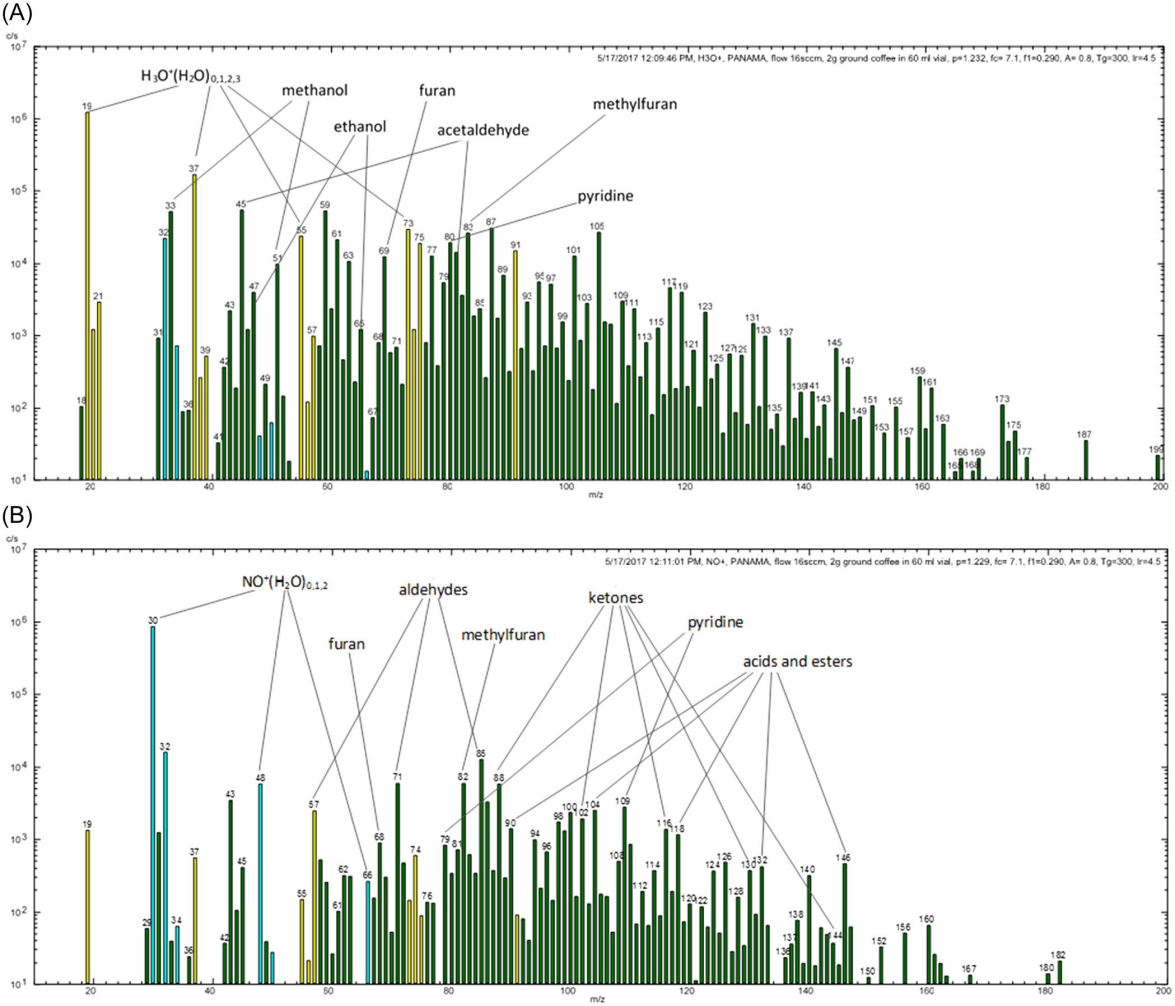
Representative SIFT‐MS full scan spectra (A) for H_3_O^+^ reagent ions and (B) NO^+^ reagent ions for the analysis of ground roasted coffee. Reproduced from Dryahina et al. (2018) with permission from Wiley. SIFT‐MS, selected ion flow tube mass spectrometry. [Color figure can be viewed at wileyonlinelibrary.com]

The extensive SIFT‐MS kinetics library created by these many SIFT and SIFT‐MS studies allows quite complex mixtures to be analyzed. A very powerful approach is to analyze such media using both H_3_O^+^ and NO^+^ reagent ions in parallel. A good example is the study of ground coffee headspace (Dryahina et al., 2018). Figure 1 shows typical *Profile 3* spectra obtained with ion peaks at almost every *m/z* value. The analytical challenge is to relate these ions to the appropriate VOC analytes. This is helped enormously by the knowledge that H_3_O^+^ reactions generally result in protonated parent molecules MH^+^ and its hydrates, whereas NO^+^ reactions generally produce parent cations M^+^ and adduct ions NO^+^M. Thus, these spectra reveal common VOCs in both spectra but with different characteristic *m/z* values (e.g., furan and methylfuran) and the appearance of groups of VOC types (e.g., acids and esters), especially in the NO^+^ spectrum. Such complex analyses would not be achieved without understanding the reaction processes outlined in previous sections. The concurrent use of H_3_O^+^, NO^+^, and O_2_^+•^ for trace gas analysis is a great triumph of SIFT‐MS.

### 3.2 Use of nitrogen carrier gas; advantages and challenges

Since the inception of SIFT‐MS, helium has been the favored carrier gas because of its exceptional chemical inertness. On injection of the reagent cations, collisional dissociation hardly occurs due to the low center‐of‐mass collisional energy, *E*_cm_, on the light helium atoms even at laboratory energies, *E*_lab_, of 10–20 eV. However, the increasing cost of helium and the looming supply problems have forced manufacturers and users of SIFT‐MS to consider nitrogen as an alternative carrier gas, especially so when long‐term monitoring of VOCs in the air is needed. It has obvious attractions in that it is relatively cheap, and supplies are essentially endless. Commercial applications using nitrogen carrier gas have shown it's applicability for environmental monitoring (Section 5.1), semiconductor airborne molecular contaminant monitoring (Section 6.3), and others, but the fundamental ion chemistry in nitrogen remains to be fully characterized.

**Figure 2:**
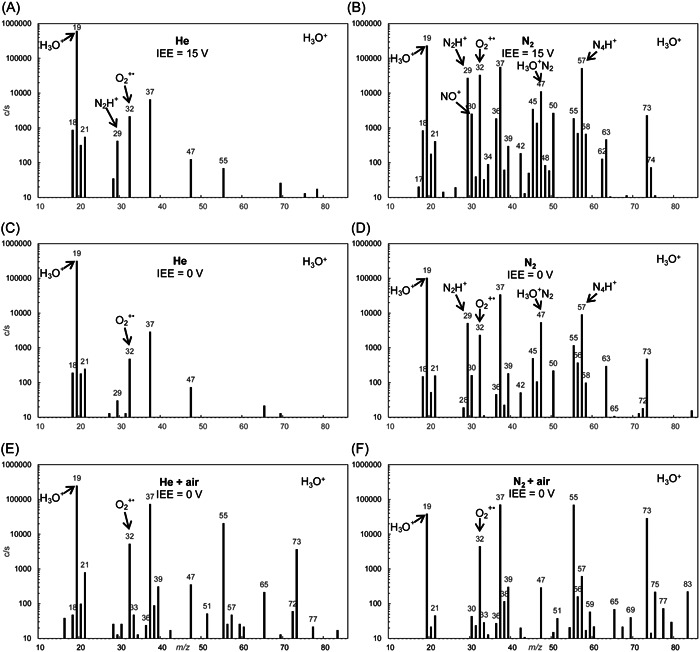
SIFT‐MS spectra obtained when H_3_O^+^ reagent ions are injected into nominally pure He and N_2_ carrier gas at ion energy electrode potentials IEE of +15 V (A, B), IEE of 0 V (C, D), and at IEE of 0 V for carrier gas containing humid laboratory air to simulate conditions used for SIFT‐MS analyses (E, F). Reproduced from Španěl and Smith (2020a) with permission from Elsevier. SIFT‐MS, selected ion flow tube mass spectrometry.

Exploratory experiments showed that injection of the reagent cations into nitrogen at the usual laboratory energies used for helium resulted in partial fragmentation of the ions because of the higher *E*_cm_. Lowering *E*_lab_ diminished the fragmentation, but this procedure can diminish the injected ion current with a consequential lowering of analytical sensitivity. This problem has stimulated a detailed comparative study of the dissociation of H_3_O^+^, NO^+^, and O_2_^+•^ as a function of *E*_lab_ (and *E*_cm_) in both He and N_2_ carrier gases at 300 K in a *Profile 3* instrument (Španěl & Smith, 2020a), using the resultant ion products by the downstream mass spectrometer as an indicator. The difference between the spectra for the injection of H_3_O^+^ into the two carrier gases is startling, as shown in Figure 2. The N_2_H^+^ and N_4_H^+^ ions are the results of the reactions of H_2_O^+•^ and OH^+^ fragment ions (that can be in electronic and rovibrationally excited states) with the abundant carrier gas N_2_ molecules. These ions may react with analyte VOCs and further complicate the spectra. Note the “cleaner” spectra at a lower injection energy and the influence of water vapor present in real samples producing an abundance of H_3_O^+^(H_2_O)_1,2,3_ and very low H_3_O^+^ ions signals.

More problematic than this reagent ion fragmentation is that H_3_O^+^ actually forms an adduct ion with N_2_ molecules, H_3_O^+^N_2_, at *m/z* 47 (Španěl & Smith, 2009). In helium carrier gas, and for the relatively small concentrations of N_2_ molecules introduced in air samples, this is not a major problem. However, in nitrogen carrier gas H_3_O^+^N_2_ production is strongly promoted, and in the presence of water molecules (humid samples) these adduct ions act as a catalyst for the production of H_3_O^+^H_2_O hydrates via the process of ligand switching:

(9) $$H_{3}O^{+}N_{2}+H_{2}O\to H_{3}O^{+}H_{2}O+N_{2}.$$

Thus, the fraction of H_3_O^+^ reagent ions in the nitrogen carrier gas is reduced and the cluster ions H_3_O^+^(H_2_O)_1,2,3_ can become the dominant ions, which seriously complicates quantitative analysis. However, increasing the N_2_ carrier gas temperature inhibits adduct ion formation as utilized in the *Voice200* instruments (Ghislain et al., 2019).

Collisional dissociation also occurs for the injection of NO^+^ and O_2_^+•^ into N_2_. The NO^+^ reagent ions partially fragment to either O^+•^ or N^+^ ions. These very energetic atomic species react with the abundant N_2_ molecules producing O_2_^+•^ ions that can be significant fractions of the NO^+^ reagent ions. Similarly, the O_2_^+•^ reagent ions dissociate to O^+•^ ions that react with the N_2_ molecules producing NO^+^ ions that can be significant fractions of the O_2_^+•^ reagent ions. In short, these collisional processes result in larger fractions of “impurity ions” than are desirable for gas phase analysis using SIFT‐MS. However, this fragmentation of NO^+^ and O_2_^+^, is less problematic than for H_3_O^+^, especially if *E*_lab_ can be reduced without lowering the analytical sensitivity too much. So, NO^+^, and O_2_^+•^ can be used as reagent ions in N_2_ carrier gas without serious complications.

Important as the above points are, they do not indicate if a N_2_‐buffered flow tube reactor can reliably provide analyses of VOCs. Specifically, can the extensive library complied with reaction kinetics largely obtained in a He‐buffered SIFT instrument be used to analyze data obtained in N_2_ carrier gas? This requires experimental investigation. Thus, a parallel study has been carried out to obtain the rate coefficients and product ion branching ratios for the reactions of H_3_O^+^, NO^+^, and O_2_^+•^ with several biogenic VOCs and monoterpenes, in both helium and nitrogen carrier gases using a *Profile 3* SIFT‐MS instrument (Španěl et al., 2022). These reactions were chosen because several reaction mechanisms are involved, including proton transfer, charge transfer, parallel hydride ion transfer, adduct ion formation, and parallel charge transfer and adduct ion formation. The conclusion is that for pure bimolecular reactions such as fast proton transfer and charge transfer, the He‐obtained kinetics data can be used with confidence for trace gas analysis by SIFT‐MS in N_2_ carrier gas. However, kinetics data for ion–molecule reactions that involve adduct ion formation must be obtained by measurements under the specific pressure (and temperature) of the N_2_ carrier gas at which gas analyses are to be taken. Clearly, more kinetics measurements are needed, also in the *Voice200* instruments, to substantiate these conclusions.

The problems associated with the use of H_3_O^+^ reagent ions in N_2_ carrier gas prompted the question as to whether another reagent ion could be used as a proton donor to analyze VOCs. The obvious point is that they must not react rapidly with either H_2_O molecules or N_2_. One such candidate is protonated ammonia, NH_4_^+^, recently introduced to PTR‐MS (Müller et al., 2020). But NH_3_ has a relatively high PA (=853.6 kJ/mol) and so NH_4_^+^ can only proton transfer to VOCs with a greater PA. Nevertheless, there are groups of VOCs with PA > PA(NH_3_), including amines and some higher‐order hydrocarbons like monoterpenes. To investigate the potential of NH_4_^+^ as a reagent cation for SIFT‐MS, the rate coefficients and product ions for its reactions with several VOCs, some with PA > PA (NH_3_) and some with PA < PA (NH_3_) have been determined in both He and N_2_ carrier gases (Swift et al., 2022). Fortunately, it is seen that NH_4_^+^ does not form adduct ions with N_2_ molecules and so H_3_O^+^H_2_O was not produced efficiently (as in reaction [9]). Adducts NH_4_^+^M were the dominant product ions in both He and N_2_ for VOCs with PA < PA (NH_3_), whereas both MH^+^ and NH_4_^+^M product ions were observed for VOCs with PA > PA (NH_3_). The conclusion is that NH_4_^+^ can be a useful reagent ion in SIFT‐MS for selected VOCs, since it does not react with H_2_O, N_2_, and He and so the NH_4_^+^ reagent ion spectrum remains relatively “pure.”

Worthy of note here is the recent development of the electrostatic reagent ion switching (*ERIS*) instrument (Španěl et al., 2019) where H_3_O^+^, NO^+^, and O_2_^+•^ reagent cations, as produced simultaneously in three separate gas discharges and “purified” in individual postdischarge source drift tubes, are selected by rapid electrostatic switching and transported into a He‐buffered drift‐tube reactor. This ERIS approach has been shown to be a potential alternative to the quadrupole filtering utilized in the *Voice200* and *Profile 3* instruments, because it realizes increased reagent cation currents, and hence increases the analytical sensitivity, of a selected ion flow‐drift tube, SIFDT‐MS, instrument (Spesyvyi et al., 2015). The weak *E‐field* in the flow‐drift reactor also inhibits the formation of the adduct ions without significantly stimulating the collisional dissociation of analyte ions.

### 3.3 Reagent anions

The great value of reagent anions is that they do react rapidly with compounds such as mineral acids, sulfur oxides, and chlorofluorocarbons (CFCs), which do not react rapidly with the available reagent cations (Hera et al., 2017). Like the cation reactions, the anion reactions involve low energy chemical ionization, which result in simple analyte ion products that can readily be identified in most cases. Thus, an immediate result was that the analysis of carbon dioxide in ambient air could be achieved using reagent O^−•^, OH^−^, and O_2_^−•^ anions. The analytical reactions proceed via ternary association to produce adduct ions, as exemplified by the reaction:

(10) $$O^{-•}+{\text{CO}}_{2}+N_{2}\to {\text{CO}}_{3}^{-•}+N_{2}.$$

It should be noted that these studies were carried out using N_2_ as the carrier gas heated to 120°C, rather than helium. Reaction (10) is slow with rate coefficient of 3.1 × 10^−28^ cm^6^ s^−1^, but easily reveals the analyte ion CO_3_^−^ at *m/z* 60 because of the high concentration of CO_2_ in the ambient atmosphere (close to 400 parts‐per‐million by volume, ppmv). Similar association reactions occur between OH^−^ and O_2_^−•^ reagent ions and CO_2_‐producing HCO_3_^−^ and CO_4_^−•^ analyte ions respectively. In exhaled breath, the CO_2_ concentration is much greater (3%–6%) and so the adduct ions are clearly seen to be formed, which can be used to determine breath CO_2_ concentration, a very valuable feature.

The bimolecular and termolecular processes involved in anion reactions are listed in a recent article (Smith et al., 2020). In some reactions, several processes can occur in parallel as it is in the case of the O^−^ reaction with acetylene (Viggiano & Paulson, 1983)

(11a) $$O^{-•}+C_{2}H_{2}\to C_{2}H_{2}O+e^{-},$$

(11b) $$\to C_{2}H^{-}+{\text{OH}}^{•},$$

(11c) $$\to C_{2}{\text{HO}}^{-}+H^{•},$$

(11d) $$\to C_{2}^{-•}+H_{2}O.$$

Here, the channels, as listed, correspond to associative detachment, deprotonation, and two variants of displacement. The process that occurs in each specific case is largely predicated by the energetics. Electron transfer occurs when the electron affinity (EA) is greater for the reactant molecule than for the electron donor. An example is the nondissociative attachment reaction of O_2_^−•^ with SO_2_F_2_ which occurs because EA(O_2_) = 0.45 eV is less than EA(SO_2_F_2_) = 0.77 eV. Similarly, the mineral acid vapors, HCl and HF, and the sulfur oxides, SO_2_ and SO_3_, are unreactive with the SIFT‐MS cations but are effectively monitored using reagent anions, especially NO_2_^−^ (Hera et al., 2017).

A recent comprehensive study of the kinetics of volatile fatty acid and aldehydes reactions using a *Voice200* instrument operating in both the anion and cation modes has been reported (Ghislain et al., 2019), which produced both rate coefficients and product ions for these VOC reactions in nitrogen carrier gas at 392 K. This interesting study has revealed much about anion reaction processes under the thermal condition that prevail in SIFT‐MS. Deprotonation is seen to occur in the reaction between O^−•^, OH^−^, and O_2_^−•^ and aldehydes producing (M−H)^−^ primary product ions, as exemplified by the acetaldehyde reaction with OH^−^:

(12) $${\text{OH}}^{-}+{\text{CH}}_{3}\text{CHO}\to {\text{CH}}_{3}{\text{CO}}^{-}+H_{2}O.$$

It is interesting to note that the product CH_3_CO^−^ ion associates with both water and acetaldehyde molecules forming CH_3_CO^−^H_2_O and CH_3_CO^−^CH_3_CHO adducts respectively (Ghislain et al., 2019), whereas it has been shown that the closed‐shell cation CH_3_CO^+^ does not efficiently hydrate (Smith et al., 2019).

In the reaction with acetic acid and other carboxylic acids, deprotonation occurs as the primary product channel:

(13) $${\text{OH}}^{-}+{\text{CH}}_{3}\text{COOH}\to {\text{CH}}_{3}{\text{COO}}^{-}+H_{2}O.$$

But for these acids, hydration of the product (M−H)^−^ ions does not occur; instead (M−H)^−^M adducts ions are formed. This seminal study (Ghislain et al., 2019) showed that most aldehydes and carboxylic acids can be analyzed by both reagent anions and cations in the *Voice200*.

A further aspect of anion chemistry in SIFT‐MS is the ability to distinguish between isomers. A good example is the structural isomers, ethyl benzene and xylene (both C_8_H_10_), which cannot be distinguished by direct mass spectrometry techniques, and which is difficult in cation chemistry. The use of OH^−^ reagent anions has enabled these isomeric compounds to be distinguished and analyzed (Allpress et al., 2019). However, the product anion in both reactions has the same molecular formula, C_8_H_9_^−^:

(14) $$\;{\text{OH}}^{-}+C_{6}H_{5}C_{2}H_{5},\left({\text{CH}}_{3}C_{6}H_{4}{\text{CH}}_{3}\right)\to {\left(C_{8}H_{9}^{-}\right)}^{*}+H_{2}O.$$

But the nascent product ion from ethyl benzene reacts with O_2_ present in the air of the sample being analyzed forming HO_2_^−^ as product ion:

(15) $$(C_{8}H_{9}^{-})*+O_{2}\to {\text{HO}}_{2}^{-}+C_{6}H_{5}{\text{CHCH}}_{2}.$$

This does not occur for the xylene‐derived C_8_H_9_^−^ product anion. It is very clear that the combination of anion and cation reagent ion chemistry is a powerful, perhaps unique tool in trace gas analysis, as provided by the *Voice200* series of SIFT‐MS instruments.

## 4 BIOMEDICAL ANALYSES; EXHALED BREATH, BACTERIAL CULTURES, AND BLOOD/PLASMA HEADSPACE

At the time of inception of SIFT‐MS (Smith & Španěl, 1996a; Španěl & Smith, 1996), the objective was to create a technique by which the analysis of air could be accomplished in real‐time, obviating sample collection into some form of vessel (bags, bottles) that can compromise the VOCs in the sample (surface condensation). A special objective was to analyze humid exhaled breath and to exploit its potential to support clinical diagnosis of disease and as a noninvasive window into human physiology. The early successes of this are described in the *Mass Spectrometry Reviews* already referred to in this paper (Smith & Španěl, 2005). The objective of the present paper is to summarize the advances made in the last decade in several areas of research and industry. This period has seen a rapid growth in the adoption of SIFT‐MS worldwide, especially the *Voice200* versions, largely for industrial applications. The much smaller number of *Profile 3* instruments have largely been exploited for research studies and this section of this paper is concerned with the efforts made to demonstrate its value in breath analysis and biogenic fluids headspace analysis.

Just a note on data acquisition. The essential measurement required to analyze a particular VOC is to identify its analyte ion and to measure its signal level (count rate) and the corresponding reagent ion signal level at the downstream analytical mass spectrometer. It is the ratio of the analyte to reagent ion signal levels that provides the concentration of the VOC in the sample. Other parameters are required for the detailed calculation, including the carrier gas pressure and the sample air/VOC gas (Španěl & Smith, 1996; Španěl et al., 2006). Breath concentrations are usually given in parts‐per‐billion by volume, ppbv, or parts‐per‐million by volume, ppmv. Indications of typical concentrations of the common breath VOCs are given in Table 1.

**Table 1:** Common volatile metabolites present in exhaled human breath.

Metabolite (MW)	Reagent ion and analyte ion (m/z)	Typical concentrations in health (ppbv)	Notes and references
Methane CH_4_ (16)	**O**_**2**_^**+•** ^CH_3_O_2_^+^ (47)	2000–30,000	Gut bacteria (Dryahina et al., 2010)
Ammonia NH_3_ (17)	**O**_**2**_^**+•** ^NH_3_^+^ (17)	300–2000^a^	Oral bacteria metabolizing urea (Smith et al., 2007; Španěl & Smith, 2018). Elevated in kidney disease (Davies et al., 2014)
Hydrogen cyanide HCN (27)	**H**_**3**_ **O** ^**+** ^HCNH^+^ (28)	1–30^a^	Bacteria in upper airways, elevated in cystic fibrosis patients infected by *Pseudomonas aeruginosa* (Gilchrist, Španěl, et al., 2015) (Smith, Španěl, et al., 2013). Elevated in smokers
Methanol CH_3_OH (32)	**H**_**3**_ **O** ^**+** ^CH_3_OH_2_^+^ (33, 51, 69)	200–1000	Gut bacteria (Smith et al., 2007). Metabolism of aspartame (Španěl et al., 2015)
Ethanol C_2_H_5_OH (46)	**H_3_O^+^** C_2_H_5_OH_2_^+^ (47, 65, 83) **NO^+^** C_2_H_4_OH^+^ (45, 63)	20−800^a^	Oral bacteria acting on sugars (Španěl et al., 2006). Gut bacteria (Smith et al., 2007)
Acetone (CH_3_)_2_CO (58)	**H**_**3**_ **O** ^**+**^ (CH_3_)_2_COH^+^ (59, 77, 95) **NO** ^**+**^ (CH_3_)_2_CONO^+^ (88) **O**_**2**_^**+•**^ CH_3_CO^+^,(CH_3_)_2_CO^+^ (43, 58)	200–1000	Catabolism of lipids, ketosis (Smith et al., 2007) Elevated due to ketogenic diet (Smith et al., 2001; Španěl et al., 2011)
Acetic acid CH_3_COOH (60)	**NO** ^**+**^ CH_3_COOHNO^+^ (90, 108)	30–60	Elevated in GERD (Dryahina et al., 2014) and CF (Smith et al., 2016a)
Isoprene C_5_H_8_ (68)	**NO** ^**+**^ C_5_H_8_^+^ (68) **O**_**2**_^**+•**^C_5_H_7_^+^, C_5_H_8_^+^ (67, 68)	10–300	Increases with age in children (Smith et al., 2010; Smith, Chippendale, Dryahina, et al., 2013). Absent in few people.
n‐Pentane C_5_H_12_ (72)	**O**_**2**_^**+•**^ C_3_H_6_^+•^, C_5_H_12_^+•^ (42, 72)	20–50	A marker of inflammation, e.g. IBD (Dryahina et al., 2013, 2018)

Abbreviation: VOC, volatile organic compounds.

^a^ These VOCs are at lower concentrations in nose‐exhaled breath (Smith, Chippendale, Dryahina, et al., 2013; T. S. Wang et al., 2008).

### 4.1 Breath analysis

This science was initiated by Linus Pauling and colleagues a half‐century ago (Teranish et al., 1972). The work over the subsequent 30 years was carried out using GC‐MS for untargeted metabolomics and using statistics to distinguish the exhaled breath VOC content of healthy persons and those suffering from various diseases. While differences have been observed, little attention has been given to specific metabolites, and statistics were used for data processing (e.g., principle component analysis, PCA). This is the approach that is currently adopted in much of breath research (Beauchamp et al., 2020). Again, in general, little attention has been given to accurate quantification of individual metabolites and the diagnostic models based on combinations of several relative VOC signals are rarely independently reproduced in other laboratories. The true value of breath analysis is surely in the recognition of volatile biomarkers of normal biological processes, pathogenic processes, or pharmacologic responses to an intervention that can be objectively measured. In the last decade or so, analysis of single breath exhalations for identified VOCs has been investigated experimentally using real‐time SIFT‐MS measurements, taking into account the possible distortion of the analyses due to ambient atmosphere exogenous VOCs (Španěl et al., 2013). This is now the major approach taken in the recent breath analysis research, largely carried out using *Profile 3* instruments. A review paper published in the *Journal of Breath Research* describes in detail the important aspects of how real‐time breath analysis is performed (Smith et al., 2014).

Much effort has been given to the measurement of breath *acetone*, principally because of its possible connection with diabetes mellitus, its concentration potentially being an indicator of glycemic control. Breath acetone can be accurately measured in single exhalations in SIFT‐MS using all three reagent ions, H_3_O^+^, NO^+^, and O_2_^+•^ (Smith et al., 2001), and this has been done in various scenarios, including populations of healthy persons (Smith et al., 2007; Španěl et al., 2007) and in type‐1 diabetic patients using a glucose clamp technique (Turner et al., 2009). However, the association with diabetes is not clear cut, as discussed in a focused paper on this issue (Smith et al., 2011). To confound this situation, it was shown by a SIFT‐MS study of single breath exhalations following a ketogenic diet taken by eight healthy individuals that their breath acetone concentrations increased variously up to five times over the subsequent 6 h postprandial (Španěl et al., 2011). These data clearly indicate that diet and natural intraindividual biological and diurnal variability result in wide variations in breath acetone concentration. The wider influence of diet on volatile breath metabolites is discussed in a focused paper (Ajibola et al., 2013).

While aspartame, methyl‐L‐α‐aspartyl‐l‐phenylalaninate, is not strictly a food, but a sweetener, its ingestion results in an increase in breath methanol in proportion to the amount of aspartame ingested. It is completely hydrolyzed in the gastrointestinal tract to aspartic acid, phenylalanine and methanol, each of which can be toxic at high levels. To investigate this, direct on‐line measurements using a *Profile 3* instrument of volatile *methanol* concentration were made in single breath exhalations from several healthy volunteers following the ingestion of aspartame (Španěl et al., 2015). The results showed that breath methanol concentrations increased in all volunteers by 3–6 times above the preingestion concentrations and then slowly decreased. This elegant study demonstrated the true value of real‐time breath VOC measurements and points the way for further studies of breath VOCs following the ingestion of other nontoxic substances. Typical concentrations of breath methanol are given in Table 1.

A misleading situation has developed in relation to breath *ammonia*, which in SIFT‐MS can best be measured using O_2_^+•^ reagent ions. The idea continues to be promoted that it can be a marker of chronic kidney disease, but this has to be challenged in view of the experimental data that is constantly emerging. The most obvious challenge is by the results of ammonia measurements in single exhalation of mouth‐exhaled and nose‐exhaled ammonia (Smith et al., 2008; T. S. Wang et al., 2008), which shows the majority of the ammonia is generated in the oral cavity by bacterial action on salivary urea. Also, there is clear evidence from much earlier SIFT‐MS studies that the consumption of a protein meal greatly increases ammonia in mouth‐exhaled breath (Smith et al., 1999). However, it should be noted that an acidic mouthwash diminishes mouth‐exhaled ammonia. A summary of the ammonia situation in chronic kidney disease, other VOCs and deuterated water has been published (Davies et al., 2014), and the real utility of breath ammonia concentration measurements in medicine and physiology has been considered (Španěl & Smith, 2018).

A more promising biomarker of disease, specifically *Pseudomonas aeruginosa*, PA, infection in cystic fibrosis, is *hydrogen cyanide (HCN)*, which has to be measured using H_3_O^+^ reagent ions, since it does not react with either NO^+^ or O_2_^+•^ (Španěl et al., 2004). This cyanide came to attention when it was shown to be emitted by cultures of PA from cystic fibrosis patients (Carroll et al., 2005). This signaled the beginning of a decade‐long study of HCN in vitro and in vivo starting with variation in HCN production between different strains of PA (Gilchrist et al., 2011), quantification of HCN and 2‐aminoacetophenone in the headspace of PA cultured under biofilm and planktonic conditions (Gilchrist et al., 2012) and the HCN concentrations in the breath of adult cystic fibrosis patients (Gilchrist et al., 2013). This work is summarized in an article in the *Journal of Breath Research* that includes a timeline of the in vivo and in vitro‐related work (Smith, Španěl, et al., 2013). The most recent study of a cohort of children with cystic fibrosis established breath HCN it as a specific biomarker of marker of early stage PA infection (Gilchrist, Belcher, et al., 2015).

*Methane*, the simplest hydrocarbon, is present in the exhaled breath of all human beings. Unfortunately, it is unreactive with both H_3_O^+^ and NO^+^, but it does slowly react with O_2_^+•^ producing the ion CH_3_O_2_^+^. Preliminary investigations showed that the O_2_^+•^/methane reaction is significantly influenced by the presence of water vapor in the SIFT‐MS reactor. So a study of the loss rate of the analyte CH_3_O_2_^+^ ion as a function of sample humidity was carried out, the results of which provided an appropriate kinetics library entry that allowed accurate analysis of methane in air and humid breath by SIFT‐MS (Španěl & Smith, 2020b). Then the methane levels, together with acetone levels as a control, were quantified in the exhaled breath of 75 volunteers, all within a period of 3 h, which shows the remarkable sample throughput rate possible with SIFT‐MS. The mean methane level in ambient air is seen to be 2 ppmv, with little spread, and that in exhaled breath for this cohort was seen to be 6 ppmv, ranging from near‐ambient levels to 30 ppmv, with no significant variation with age and gender; see Table 1.

The most abundant breath hydrocarbon other than methane is *isoprene* (C_5_H_8_), which can be analyze in SIFT‐MS using all three reagent cations, but its concentration is best measured using NO^+^ to avoid ion overlaps. It has been measured in single exhalations of breath from cohorts of healthy people since the early days of SIFT‐MS (Turner et al., 2006) and in the exhaled breath of 200 healthy pupils within the age range 7–18 years (Smith et al., 2010). The interesting result that emerges from the latter study is the breath isoprene peaks at puberty, around 13 years of age, which has not yet been explained from a physiological viewpoint. It is interesting to report that isoprene was shown to increase in exhaled breath during laparoscopic surgery (Boshier et al., 2011) using a *Profile 3* instrument located in the operating theatre.

Quantification of the alkane *n‐pentane* (C_5_H_12_) in exhaled breath is of even greater interest and utility since it is related to inflammation in the body and is thus potentially a biomarker of inflammatory bowel disease (IBD). The analysis of n‐pentane is achieved using O_2_^+•^ reagent ions, as mentioned in Section 3.1. To investigate this, a study has been carried out of the VOCs in the exhaled breath of patients suffering from IBD, comprising cohorts with Crohn's disease (CD) and ulcerative colitis (UC) (Dryahina et al., 2013). This study was later followed up by a further study of n‐pentane and other VOC, including carboxylic acids, in the breath of patients with CD and UC that corroborated the findings of the first study (Dryahina et al., 2018). Breath samples were measured both on‐line for immediate analysis and collected into Nalophan bags, which were then quantitatively analyzed using SIFT‐MS off‐line. It was seen that the median concentration of pentane was elevated in the samples from IBD patients compared to those of the healthy controls. However, the absolute median pentane concentrations in the bag samples were about a factor of two lower than those in the directly analyzed single exhalations—a good illustration of the dilution of VOCs in breath samples collected into bags (Dryahina et al., 2018). The relative concentrations of pentane and these other VOCs weakly correlate with simple clinical activity indices.

Trace amounts of *alkene* vapors in exhaled breath have been reported to be different in the breath of IBD patients in comparison with healthy controls (Michalcikova et al., 2016). Thus, a detailed SIFT, study of the reactions of H_3_O^+^, NO^+^, and O_2_^+•^ with an homologous series of alkenes (from 1‐hexene to 1‐decene) and with additional isomers of heptene (*trans*‐2‐heptene and a mixture of *cis*‐ and *trans*‐3‐heptene) has been carried out (Michalcikova et al., 2018). The rate coefficients and product ion branching ratios of the reactions of H_3_O^+^ and NO^+^ ions were also obtained, as required for accurate SIFT‐MS quantification of alkenes in exhaled breath with the aim of establishing their potential clinical use.

**Figure 3:**
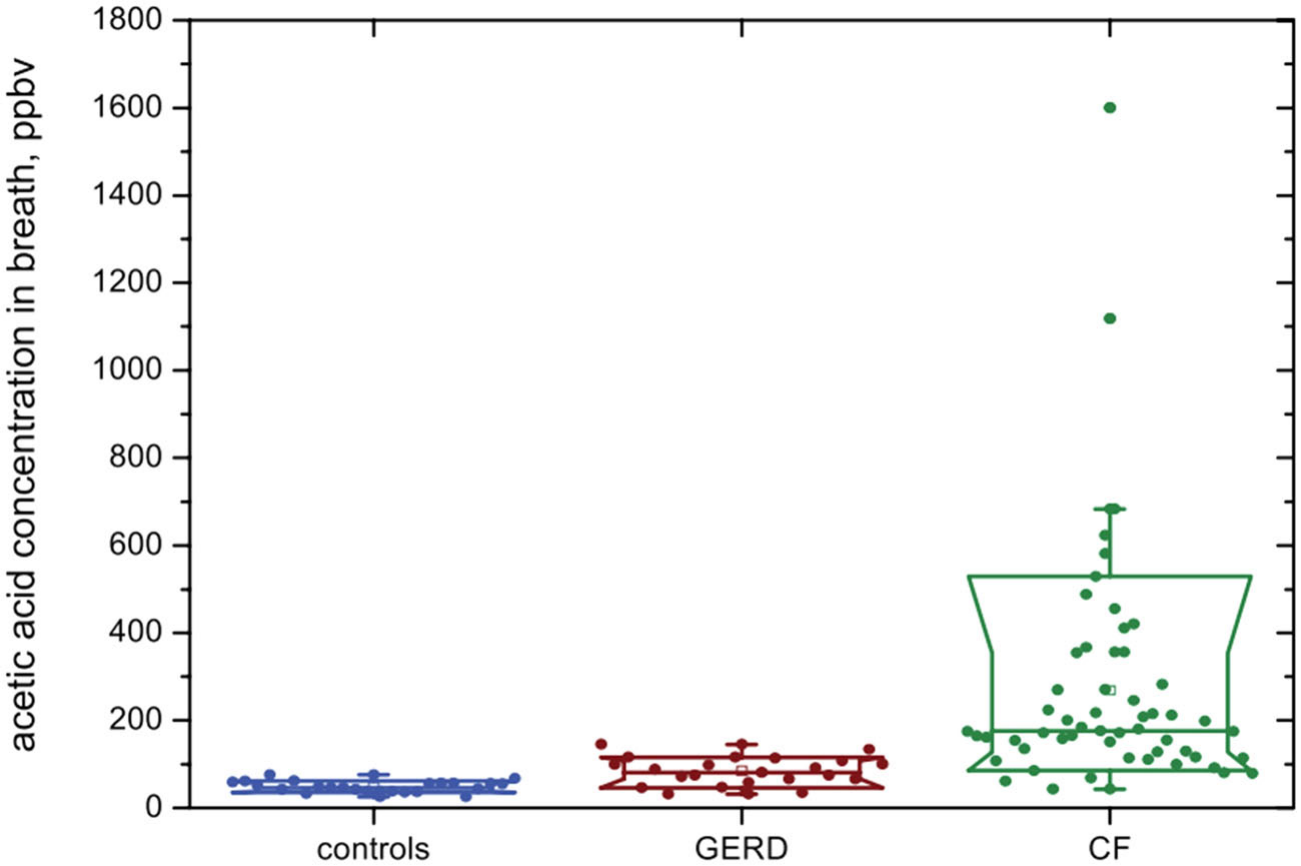
Concentrations of acetic acid in exhaled breath of healthy controls and patients with gastro‐esophageal reflux disease (GERD) (Dryahina et al., 2014) and patients with cystic fibrosis (CF) obtained using SIFT‐MS. In addition to the individual data points, the box‐and‐whisker plots are included to indicate the 10th, 25th, 50th, 75th, and 90th percentiles. Reproduced from Smith et al. (2016b) with permission from IOP publishing. [Color figure can be viewed at wileyonlinelibrary.com]

Motivated by a real clinical need, an experimental study was initiated to discover any VOCs present in exhaled breath that could be used as biomarkers of gastro‐esophageal reflux disease (GERD), one of the most common causes of chronic cough. Thus, the VOCs emitted from pork tissue samples exposed to a challenge by artificial gastric fluid were analyzed by SIFT‐MS supported by GC‐MS (Dryahina et al., 2014), which indicated that the only VOC that significantly increased was *acetic acid*. The end expiratory concentration of acetic acid measured by SIFT‐MS in mouth‐exhaled breath of 22 GERD patients (median 85 ppbv) were significantly higher than in breath of a control group (median 48 ppbv). Thus, it was suggested that acetic acid may be useful for noninvasive diagnostics of airways acidification in GERD, as illustrated in Figure 3. Further to this GERD study, while investigating VOC in the breath of patients with cystic fibrosis (CF) it was discovered that acetic acid is significantly elevated in the exhaled breath of the CF patients compared to that of healthy controls (Smith et al., 2016b) and much greater, on average, than the breath acetic acid than for the GERD patients, also shown in Figure 3. It was speculated that the cause of elevated breath acetic acid may be a decreased *pH* of the mucus lining the CF airways and that it could serve as a noninvasive indicator of the acidity of the CF airways mucosa (Španěl, Sovová, et al., 2017).

It would be remiss not to comment on the SIFT‐MS work carried out to search for breath VOCs related to cancer. Most of the previous work in this area involved untargeted observations using GC‐MS to analyze exhaled breath and resulted in long lists of candidate VOCs suggested as possible biomarkers, usually in combinations of several compounds related to various types of cancer. This work was extensively reviewed by a group of workers in breath research (Haick et al., 2014). Analytical methodologies are continuously developing together with metabolomic approaches to identify such biomarkers (Lubes & Goodarzi, 2018). *Profile 3* SIFT‐MS studies of exhaled breath in relation to cancer screening based on quantification of concentrations of targeted compounds have been carried out (Kumar et al., 2013). The SIFT‐MS quantification of VOCs in the exhaled breath from patients with esophagogastric cancer (OGC), revealed that hexanoic acid, phenol, methyl phenol, and ethyl phenol were significantly involved.

A subsequent *Profile 3* SIFT‐MS study (81 OGC patients, 33 gastric adenocarcinoma patients, and 129 controls) showed that 12 VOCs (pentanoic acid, hexanoic acid, phenol, methyl phenol, ethyl phenol, butanal, pentanal, hexanal, heptanal, octanal, nonanal, and decanal) were present at significantly higher concentrations (*p* < 0.05) in the cancer groups than in the noncancer controls. A risk‐prediction model was constructed that could discriminate OGC and gastric adenocarcinoma with areas under the constructed receiver operating characteristic (ROC) curve of 0.97 for all compounds and 0.83 for a subset of five compounds. This 5‐VOC model (butyric acid, pentanoic acid, hexanoic acid, butanal, and decanal) was then tested on targeted SIFT‐MS breath analyses of a larger group (163 esophagogastric cancer patients and 172 controls), confirming its good accuracy for the diagnosis of OGC with an area under the ROC curve of 0.85, sensitivity of 80% and specificity of 81% (Markar et al., 2018).

Encouraged by this success, a dedicated breath analysis center has been established in London, UK, equipped with a *Voice200* TD‐SIFT‐MS, multiple GC‐TOFMS, and a GCxGC‐MS instruments. A comprehensive workflow for on‐line and off‐line breath sampling has been detailed and published in *Nature Protocols* (Belluomo et al., 2021) to standardize breath analyses using SIFT‐MS, allowing 50 breath samples to be analyzed and interpreted in 3 h.

SIFT‐MS is usually used for targeted breath analyses; however, some studies attempt to process untargeted full‐scan data for pattern‐based classification as done, for example, example in a recent study of asthma (Stefanuto et al., 2020). While the results of these studies are promising, without quantification of identified metabolites, their independent verification is less straightforward than for targeted studies.

### 4.2 Bacterial culture emissions

**Figure 4:**
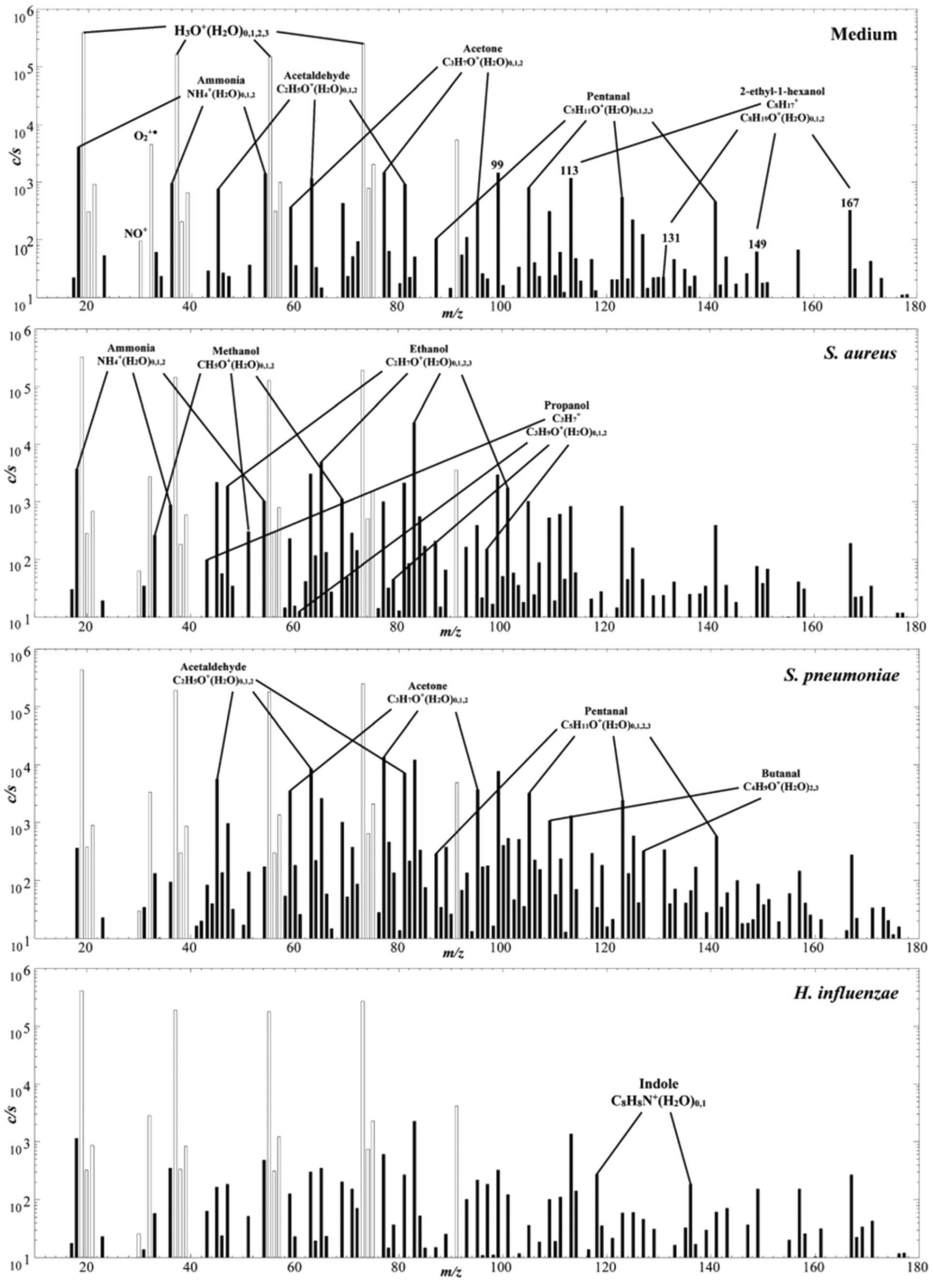
Example full scan (FS) mass spectra (ion counts‐per‐second, *c/s*, plotted against ion mass‐to‐charge ratio, *m/z*) obtained using H_3_O^+^ reagent ions for the *Profile 3* analysis of the headspace of neat culture medium and cultures of *Staphylococcus aureus*, *Staphylococcus pneumoniae*, and *Haemophilus influenzae*, as specified. Reproduced from Chippendale et al. (2014b) with permission from the Royal Society of Chemistry under the Creative Commons License.

The rationale for including such measurements in association with breath research is the tantalizing possibility that the VOC emissions from pulmonary and airways bacteria may be detected in exhaled breath, thus providing a noninvasive diagnosis of such infections. In this regard, SIFT‐MS has been utilized to identify VOCs from in vitro cultures of specific bacteria. Already in this review, VOC emissions from *Pseudomonas aeruginosa* (PA) have been discussed, in particular the release of HCN. Following this important work, SIFT‐MS analyses were conducted to identify and quantify the VOCs emitted by clinical isolates of the respiratory pathogens *Staphylococcus aureus* (SA), *Streptococcus pneumoniae* (SP), and *Haemophilus influenzae* (HI) and the medium in which they were cultured (Chippendale et al., 2014b). Sample *Profile 3* spectra are shown in Figure 4. Six VOCs, mainly alcohols, ketones and aldehydes, were found to be elevated in the headspace of SA cultures and eight VOCs were elevated in the SP cultures. Only indole and ethanol were somewhat elevated in the headspace of some of the HI cultures. It was postulated that high concentrations of ethanol and acetaldehyde in exhaled breath above those for healthy people may provide indicators of airways infection by SA and SP. The clinical value of this work needs to be substantiated by further work on the VOC emissions from these respiratory pathogens.

Subsequently, this work was extended to investigate the VOCs emitted by in vitro cultures of the common respiratory fungus *Aspergillus fumigatus* (AF), again using real‐time analysis using SIFT‐MS (Chippendale et al., 2014a). It was seen that copious amounts of ammonia and the organosulfur compounds methanethiol, dimethyl sulfide, and dimethyl disulfide were released by AF fungus cultures, which may be sufficient to allow noninvasive detection of the AF in the airways of infected patients by breath analysis. Although it was seen that AF efficiently absorbs and metabolizes acetaldehyde, butanal and pentanal from the supportive medium (brain‐heart infusion broth), preliminary studies of the VOCs emitted by cocultures of AF with PA, SA and SP revealed that the biomarker HCN (for PA) is not compromised by the presence of AF, and the organosulfur compounds (for AF) are not compromised by the presence of SA or SP, which bodes well for breath analysis studies of these pathogens.

Further to this work, a study was carried out to characterize the VOCs produced by genotypically diverse strains of the *Stenotrophomonas* genus to evaluate their potential as breath biomarkers of lung infection by this bacterium (Shestivska, Dryahina, et al., 2015). VOCs emitted from 15 clinical and five environmental strains belonging to different genogroups of *Stenotrophomonas maltophilia* (*n* = 18) and *Stenotrophomonas rhizophila* (*n* = 2) cultured in Mueller‐Hinton Broth (MHB) liquid media were analyzed by SIFT‐MS supported by GC‐MS. Several VOCs were detected in high concentration, including ammonia, propanol, dimethyl disulfide propanol, and dimethyl disulfide, but the rates of production of the VOCs by the genotypically‐distinct strains were very similar. All in vitro cultures of both the *Stenotrophomonas* species were characterized by efficient production of two isomers of methyl butanol, which is absent in other pathogens, including PA. These data indicate that methyl butanol isomers may be exhaled breath biomarkers of *S. maltophilia* lung infection in patients with CF.

*Escherichia coli JM109* is a classic *E. coli* strain for routine cloning and plasmid maintenance. The preliminary SIFT‐MS work involved measurements of the VOCs emitted by this bacterium cultured in the commonly used media Dulbecco's modified Eagle's medium (DMEM) and lysogeny broth (LB) using a *Profile 3* instrument, as a step towards the real‐time, noninvasive monitoring of accidental infections of mammalian cell cultures (Chippendale et al., 2011). In DMEM, copious amounts of ethanol, acetaldehyde, and hydrogen sulfide were produced; in LB, ammonia was the major volatile product. Maxima occurred in the ethanol and acetaldehyde production, a reflection of the reduction of glucose from the DMEM by the vigorous *E. coli* cells. A maximum in the hydrogen sulfide level was an indication of the loss of the sulfur‐bearing amino acids from the DMEM. When large quantities of ammonia were produced from DMEM, it was deduced that it had become inadvertently infected with the bacterium *C. testosterone*. The results of this preliminary study suggest that monitoring volatile compounds might assist in the early detection of bacterial infection in large‐scale bioreactors, which would be of great commercial value.

### 4.3 Plasma/blood headspace

**Figure 5:**
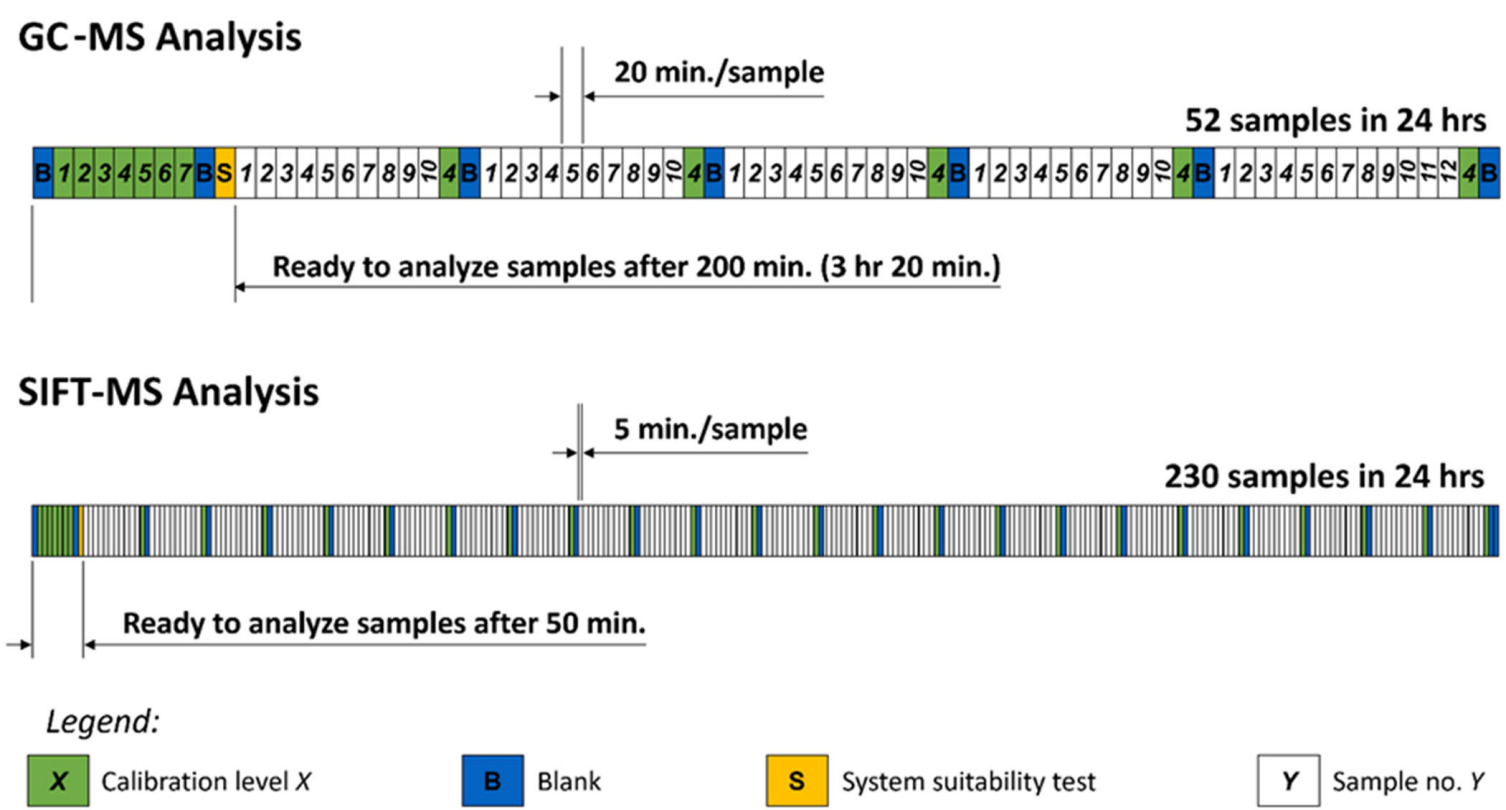
Daily sample schedules for GC‐MS and SIFT‐MS analyses of porcine plasma samples. Note that the full quality control (QC) sequence takes only 50 min. for headspace‐SIFT‐MS, which is four times faster than the same QC schedule with GC‐MS and the number of samples per day is also four times greater. GC‐MS, gas chromatography–mass spectrometry; SIFT‐MS, selected ion flow tube mass spectrometry. [Color figure can be viewed at wileyonlinelibrary.com]

SIFT‐MS blood and plasma headspace analyses so far have been a minority application, as exemplified by quantification of aldehydes in various mouse tissues and plasma (Ross et al., 2013). Recently, parallel GC‐MS and SIFT‐MS analyses of cyclohexanone and cyclohexanol in porcine plasma have indicated that, after calibration, the quantificational results are comparable, but SIFT‐MS has four times higher throughput (Hastie et al., 2021). Analytical speed can be very important, for example, in diagnosis poisoning using agricultural pesticides. The time to analyze a sample from set‐up, analysis of blanks, and standards is also much faster with SIFT‐MS than with GC‐MS (see Figure 5).

## 5 ENVIRONMENTAL APPLICATIONS

SIFT‐MS is having an increasing impact on environmental monitoring due to the enhanced sensitivities of modern commercial instruments that typically employ nitrogen carrier gas. Thus, SIFT‐MS is now considered to be a valuable, low‐cost, and easy‐to‐use alternative for atmospheric trace gas analysis of such complex mixtures, even of isomers, at a varying humidity (Lehnert, Behrendt, et al., 2020).

### 5.1 Ambient atmospheric monitoring and research

Twelve years ago, continuous ambient monitoring by SIFT‐MS was demonstrated, targeting benzene, 1,3‐butadiene, ethanol, ethene, and toluene in a light industrial area in Christchurch, New Zealand (Prince et al., 2010). A laboratory study determined the linearity range for each compound and showed that an instrument detection limit of 6.4 parts‐per‐trillion by volume, pptv, was achieved for 1,3‐butadiene with a 32‐s measurement time. However, benchmarking with the gold‐standard measurement technique (GC‐MS) for VOCs is a necessary consideration when introducing a new technology, such as SIFT‐MS. Since high‐sensitivity, direct analysis of air is challenging for GC‐MS, this was conducted via canister‐based sampling (Langford et al., 2014). The GC‐MS analysis was conducted according to the United States Environmental Protection Agency (US EPA) TO‐15 method (EPA, 1999). Comparability for a subset of hydrocarbons and chlorinated hydrocarbons between SIFT‐MS and GC‐MS was good, except in hydrocarbon‐rich soil gas samples obtained near fuel stations for which the hydrocarbon analyte ions overlapped with those for the chlorinated species.

SIFT‐MS has been adopted by the Wolfson Atmospheric Chemistry Laboratory (WACL) at the University of York (UK), which is part of the UK National Centre for Atmospheric Science (NCAS). In 2016 and 2017 a *Voice200* SIFT‐MS instrument was deployed in Beijing, China to participate in the Air Pollution and Human Health in a Chinese Megacity program (Shi et al., 2019). This was the first remote deployment of its type for SIFT‐MS instrumentation, with unattended operation for two 6‐week deployment periods (November–December 2016 and May–June 2017), including automated calibration on a 3‐day cycle. Air was sampled at a height of 102 m and delivered to the SIFT‐MS instrument located in a hut at ground level. Most SIFT‐MS VOC measurements are currently unpublished. Nevertheless, SIFT‐MS measurements of isoprene were used to fill gaps in dual‐channel gas chromatography‐flame ionization detector (DC‐GC‐FID) data, albeit with a correction made for the reduction in isoprene levels at higher elevation when measured with SIFT‐MS compared to DC‐GC‐FID, which were sampled at 8 m (Bryant et al., 2020). In addition to measurement of VOCs in Beijing, an intercomparison of instrumentation for nitrous acid (HONO) analysis was conducted (Crilley et al., 2019). HONO is a challenging compound to measure in the field, especially when there is significant air pollution, but is of great importance in understanding the OH radical abundance; it can be analyzed using SIFT‐MS (Smith et al., 1999; Sovová et al., 2010). Although SIFT‐MS did not participate in the full HONO intercomparison, from a limited measurement period it was evident that the technique correctly followed the trends (through high correlation with other techniques), while a PTR‐TOF‐MS instrument could not detect HONO using the same reagent ion (H_3_O^+^).

In March 2019, a *Voice200* SIFT‐MS instrument was deployed in Hanoi, Vietnam's second most populous city, for a spring season measurement campaign. Alongside a DC‐GC‐FID instrument, it measured 20 organic and inorganic compounds (Hien et al., 2022). The use of SIFT‐MS in Hanoi, and PTR‐TOF‐MS in Ho Chi Minh City, enabled real‐time ambient analysis of oxygenated VOCs to be conducted for the first time in Vietnam. This is an important addition because the oxygenated VOCs contribute over 25% of the VOCs and are important in ozone formation. A recent laboratory study comparing different techniques for analyses of atmospherically significant oxygenated VOCs (Borras et al., 2021) demonstrated that SIFT‐MS measurements were consistently the most accurate at various dilutions, under both dry and humid conditions.

More recently, SIFT‐MS instrumentation has been incorporated into mobile laboratories to monitor VOCs and inorganic compounds in ambient air, both spatially and temporally (Wagner et al., 2021). Sub‐ppbv detection limits were achieved for 12 of the 13 analytes (NO_2_ was the exception at 2.5 ppbv) using a 2.5‐s time resolution. Mobile monitoring is a necessary development for improvement in VOC source inventories, since motor vehicle emissions diminish and—proportionately speaking—VOCs dominate that are derived from volatile chemical products used in both industrial and domestic applications (Lewis et al., 2020). This has contributed to a broadening of SIFT‐MS application, wherein it is used to quantify VOC emission rates from personal care products in actual product usage scenarios within the home. Recently, SIFT‐MS has been applied to nonaerosol products (Yeoman et al., 2020) and facial moisturizers (Yeoman et al., 2022) causing increased doses of VOCs inhaled by the user compared to typical daily doses inhaled in indoor air. Cleaning and cooking are other sources of volatile emissions. Research is in its early days, but real‐time analysis using SIFT‐MS offers significant benefits in understanding the contribution of these volatiles to both personal exposure indoors and emissions inventories. One area that would benefit from investigation is the interior air of aircraft cabins containing semi‐volatile molecules such as organophosphate esters flame retardants, based on a recent kinetics study by Ghislain et al. (2022).

Research conducted in Korea has devoted attention to SIFT‐MS in continuous monitoring applications. In an effort to increase responsiveness to air pollution incidents and to overcome the slow time to obtain analytical results by conventional grab sampling, the Korean Institute for Oceanic Science and Technology (KIOST) has published a 60‐compound analytical method with a cycle time of 3.2 min (Son et al., 2018). They achieved good linearity in concentration over the calibration range from 0.174 to 100 ppbv, with repeatability (measured using relative standard deviation, %RSD) less than 10%, and sub‐ppbv limits‐of‐detection, LODs, across the wide range of chemical functionalities (including amines, hydrocarbons, organosulfur compounds, and volatile fatty acids). Several of these authors also utilized SIFT‐MS in mobile laboratories to investigate diurnal variation of VOCs (alongside carbon dioxide and ozone) at representative urban and harbor sites in Busan, Korea, from January to April 2019 (Hwang et al., 2020). The mobile laboratory enabled measurements to be made near region‐specific sources. A suite of 53 VOCs, including alkanes, alkenes, aromatics, alcohols, carboxylic acids, esters, aldehydes, ketones, and halogenated compounds, were measured using SIFT‐MS. Carbonyl compounds accounted for 60% and 70%, respectively, of the ozone formation potential at urban and harbor sites. Formaldehyde showed the highest ozone production per concentration index (OPCI). This study indicates potential SIFT‐MS applications in future studies focused on ozone formation.

Thermal desorption of VOCs in combination with SIFT‐MS analysis has been tested in research laboratories previously (Hryniuk & Ross, 2009; Sovova et al., 2019). However, a fully integrated commercial TD‐SIFT‐MS system has become available recently (Langford et al., 2021). In environmental applications this instrument achieves similar detection limits to TD‐GC‐MS, but it can analyze up to 15 sorbent tubes per hour, compared to just three using TD‐GC‐MS.

SIFT‐MS has also been used in more fundamental atmospheric research by several groups at Mines Douai and the University of Lille to study fundamental surface‐air adsorption and desorption kinetics of VOCs interacting with atmospheric dusts (Romanias et al., 2016; X. J. Wang et al., 2020; Zeineddine et al., 2017, 2018) This research supports improved modeling of the fate of VOCs in the atmosphere, which is an important consideration for secondary organic aerosol (SOA) formation, a significant environmental and health hazard (Lewis et al., 2020). SIFT‐MS has been used to monitor formation and decay of various VOCs in smog chambers (Li & Cocker, 2018; L. J. Li et al., 2017; W. H. Li, Li, et al., 2018) to understand the formation of SOAs. Similarly, SIFT‐MS has been used to evaluate the role of ammonia in SOA formation (K. W. Li, Chen, et al., 2018).

### 5.2 Odor analysis

SIFT‐MS research of odiferous compounds has received considerable attention in the last decade. Note that some compounds targeted for ambient monitoring, as mentioned above (Son et al., 2018), are also important odorants. Examples of such work include SIFT‐MS odor measurements at intensive animal facilities (laying hens, broiler chickens, and pigs) that agree well with parallel TD‐GC‐MS measurements (Van Huffel et al., 2012) and enabled rapid matching of odor profiles to library patterns using similarity coefficients (Heynderickx et al., 2012, 2013). SIFT‐MS measurements were then directed to improvements in bio‐filtration technology. Foundational work on partitioning coefficients (Bruneel et al., 2016; Heynderickx et al., 2014) and sorption properties (Walgraeve et al., 2015) became the basis for subsequent research of the fundamentals of mass transfer (Bruneel, Walgraeve, Dumortier, et al., 2018; Volckaert et al., 2016) and evaluation of new media (Bruneel, Walgraeve, Mukurarinda, et al., 2018; Bruneel et al., 2020; Volckaert et al., 2016;) utilizing the real‐time capability of SIFT‐MS.

SIFT‐MS was also used to monitor odor emissions from intensively farmed broiler chickens to reduce the litter odors through modified diet (Sharma, Choct, et al., 2017) and to understand the microbial origins of increased odor during *Clostridium perfringens* infection (Sharma, Keerqin, et al., 2017).

Recently, a *Voice200* instrument was used to investigate odor sources at a gelatin factory (Langford et al., 2018) and a large wastewater treatment plant (WWTP) (Langford et al., 2020). Both studies demonstrated that the ability to analyze a wide variety of odorants (aldehydes, amines, organosulfur compounds, volatile fatty acids, etc.) facilitated development of odor “fingerprints” for various odor sources in the facilities. In the gelatin factory study, SIFT‐MS was subsequently used to assess the effectiveness of odor neutralization technology based on ultraviolet photolysis. The WWTP study evaluated SIFT‐MS odor analysis versus gold‐standard olfactometry by a trained sensory panel. SIFT‐MS has the potential to provide improved instrument‐based sensory analysis (compared to sensors), though further research and wider application is required.

### 5.3 Water and soil analysis

Korea water has utilized SIFT‐MS for the analysis of industrial wastewater (S. H. Lee et al., 2020), which is potentially more challenging for SIFT‐MS than studies in air. This study demonstrated that a novel, prototype automated headspace approach was suitable for deployment at site, with simple maintenance requirements. The limits of detection for 22 out of 28 compounds met or exceeded the requirements set by the Korean Ministry of Environment.

Automated analysis of water headspace in the Anatune Laboratory (Cambridge, UK) has demonstrated even better limits of quantitation (LOQs) in the sub‐μg L^−1^ range for benzene, toluene, ethyl benzene and xylene (BTEX) and 1.5 μg L^−1^ for chloroform using direct injection of headspace (Perkins et al., 2018). A subsequent validation study from the same laboratory (Perkins & Langford, 2021b) targeted 17 VOCs and achieved LOQs of 10 μg L^−1^ or better. Improving detection limits for direct aqueous headspace analysis is an area of ongoing study. The robustness of SIFT‐MS analysis to sample humidity, leads to simplified sample preparation compared to most GC methods, and coupled with high sample throughput these are significant advantages of SIFT‐MS compared to traditional analytical techniques.

Soil analysis using SIFT‐MS has received little attention to date, but it has potential to facilitate rapid screening of volatile pollutants in soil samples, even on site using mobile laboratories. Quantitative analysis is, however, a challenge with static headspace due to different soil types and moisture levels affecting partitioning. Hence, a preliminary investigation has been made with SIFT‐MS of the widely used (with GC) methanolic extraction procedure. The main limitation of this sample preparation approach with SIFT‐MS is that only the NO^+^ reagent ion can be used, due its low reactivity with methanol (Perkins & Langford, 2021a). Nevertheless, good results have been obtained when targeting common soil pollutants from petroleum (BTEX).

### 5.4 Sustainability

SIFT‐MS is also used in ecologically focused research. The applicability of SIFT‐MS has been demonstrated by the analysis of environmental microplastic residues, pollutants of increasing concern—an area to date principally researched using the established methods of thermogravimetric analysis (TGA)—(Mansa & Zou, 2021; Peñalver et al., 2020) or pyrolysis‐GC‐MS analysis (Hermabessiere et al., 2018). SIFT‐MS is complementary to the thermal analysis techniques, because sensitive analysis can be conducted at significantly lower temperatures minimizing polymer degradation, and hence it enables volatile components and degradation products to be identified and quantified. Application of PCA to SIFT‐MS FS spectra enabled qualitative identification of recovered plastics as polypropylene or low‐density polyethylene (LDPE) (La Nasa et al., 2021), and identification of volatile residues in environmental samples enabled quantitative analyses to be performed. Even though the number and variety of samples investigated was limited, the results indicated that the extent of oxidation might be determined from the relative concentration of alcohols, carboxylic acids, aldehydes, and ketones (for even carbon numbers from 2 to 8). The value of rapid SIFT‐MS analysis was demonstrated further as part of a wider protocol for comprehensive micro plastics identification (Castelvetro et al., 2021). In particular, SIFT‐MS enabled rapid concentration determinations to be obtained for toxic volatile degradation products. A further interesting SIFT‐MS study characterized the response of edible and nonedible plants to crushing by releasing signaling VOCs (Kim et al., 2019). This response was greatest in edible plants that experience crushing and exposure to human breath.

SIFT‐MS has also been used to measure very low levels of VOCs from soil, leaves, and leaf litter. After modifying a commercial *Voice200* instrument to maximize sensitivity (Lehnert, Behrendt, et al., 2020), the benefits of SIFT‐MS analysis over PTR‐MS analysis for differentiation of isoprene and 2‐methyl‐3‐buten‐2‐ol were demonstrated both in ideal systems as well as from trees (Lehnert, Perreca, et al., 2020).

SIFT‐MS has also been utilized in alternative energy research. It has been applied to real‐time detection of VOCs produced through electrochemical reduction of carbon dioxide (Lobaccaro et al., 2018; Mandal et al., 2018), an approach to reduce CO_2_ emissions.

A commercial landfill gas analysis service in New Zealand was running from 2005 to 2018. Landfill gas contains toxic siloxanes (Langford et al., 2013), which are also of industrial significance due to their oxidation to silica in gas turbines that can increase maintenance requirements and reduce service life. Recently, *Profile 3* SIFT‐MS was used in Prague to analyze trace volatile impurities in biogas (methane) (Knizek et al., 2018) and *Voice200* SIFT‐MS was optimized for analyses of BTEX impurities in this fuel (Allen et al., 2019).

## 6 WORKPLACE SAFETY AND INDOOR AIR QUALITY

VOCs are important contributors to workplace exposure and indoor air quality. People can be exposed to VOCs for long periods each day in these environments, which can be adversely chronic. Two applications in which the SIFT‐MS technique is making significant impact in industry are briefly described: quantification of hazardous substances (including fumigation chemicals) in shipping containers, and ultra‐sensitive 24/7 monitoring of volatiles in semiconductor cleanrooms.

### 6.1 Research of indoor air quality and pollution

The past few years have seen renewed interest in SIFT‐MS in the workplace exposure research, such as studies on exposure to airborne solvents in the collision repair industry (Keer et al., 2018) and fumigation chemicals in shipping containers (Hinz et al., 2020; Hinz et al., 2022) (also see Section 6.2). A SIFT‐MS‐based method for identifying and quantifying leakage from closed system drug‐transfer devices has been developed (Doepke & Streicher, 2021), and SIFT‐MS was very recently validated for real‐time monitoring of a new dosing system for reference gases (Kaus et al., 2022). Further, an interesting SIFT‐MS application has been described for simultaneously measuring VOCs in vehicle cabin air (Zhu et al., 2017). Previous approaches entailed separate analyses of several VOCs by GC and of volatile aldehydes by HPLC. Now rapid, on‐site vehicle interior air quality (VIAQ) testing using SIFT‐MS could be achieved, which has potential commercial relevance to automotive manufacturers.

### 6.2 Fumigant detection

Shipping containers are typically airtight due to welded steel walls and rubber seals on the access doors. Whilst this means that they effectively protect the goods that they are carrying from damage, it also ensures that fumigation chemicals (fungicides and pesticides to prevent harmful organisms crossing borders) and volatiles emitted from the contents are trapped and accumulated in the confined space. This means that unpacking a shipping container at its destination can be a hazardous task (Baur et al., 2010; Hinz et al., 2020, 2022).

Strategies for protecting workers from these hazards include mechanical ventilation of each container, testing containers using simple photoionization detector devices, colorimetric tubes, metal oxide‐based sensors, and portable infrared spectrometers, all of which have their limitations. Routine laboratory analysis is generally impractical in terms of cost and turnaround time as the results are needed at low cost and as quickly as possible to move containers through the acceptance process. Moreover, the chemical diversity of the fumigants based on polarity and ionization energy means that multiple GC analyses are required to report the full suite of volatiles. However, SIFT‐MS has proved effective in this application (Hinz et al., 2020, 2022; Milligan et al., 2007), especially when coupled with powerful risk assessment algorithms that identify which containers should have chemical testing conducted. The SIFT‐MS reagent cations (Section 3.1) can detect all common fumigants listed in Table 2 except sulfuryl fluoride, which is detected using reagent anions (Section 3.3). Some operators also target toxic industrial chemicals (TICs) such as benzene and toluene. Samples are usually collected in Tedlar® sample bags and analyzed by SIFT‐MS in less than 2 min. The analytical method is factory calibrated and due to an automated instrument performance check procedure, it requires only annual recalibration. Simple pass/fail reporting is provided instantaneously on the user interface, enabling instant decision making for nontechnical users.

**Table 2:** Fumigants analyzed by SIFT‐MS.

Compound name	Trade or common name	Formula	CAS number	Comment
Bromomethane	Methyl bromide	CH_3_Br	74‐83‐9	
1,2‐Dibromoethane	Ethylene dibromide	C_2_H_4_Br_2_	106‐93‐4	
Trichloronitromethane	Chloropicrin	CCl_3_NO_2_	76‐06‐2	Added to methyl bromide as odorant
Hydrogen cyanide		HCN	74‐90‐8	
Sulfuryl fluoride	Vikane®	SO_2_F_2_	2699‐79‐8	
Phosphine		PH_3_	7803‐51‐2	
Ethylene oxide		C_2_H_4_O	75‐21‐8	
Formaldehyde		CH_2_O	50‐00‐0	Most commonly outgassing from product

Abbreviation: SIFT‐MS, selected ion flow tube mass spectrometry.

### 6.3 Semiconductor industry

Advances in semiconductor manufacturing have resulted in much narrower gate widths and more transistors per chip. Narrower gate widths mean that the impact of contamination is greatly enhanced and yields are dramatically reduced, but currently there is no option but to continue to use chemical reagents in the fabrication or cleaning processes (Den et al., 2020). Further, contamination issues can arise due to spillages, leaks, or breakthrough in air filtration systems. To summarize, controlling airborne molecular contaminants (AMCs) is essential if product adverse quality issues are to be avoided, and any lowering in air quality must be detected rapidly and at very low contamination levels (ppbv concentrations and below). Moreover, employees benefit from minimized exposure to toxic compounds.

Although there is a very wide range of potential AMCs in the production environment, a small subset is of greatest concern. In addition to organic solvents, these include acidic (HF, HCl, SO_2_) and basic (NH_3_) permanent gases. The conventional approach has been to analyze organics using TD‐GC‐MS and the acidic and basic gases analyzed using either ion chromatography or spectroscopic methods (i.e., multiple instruments are required and several of these are off‐line) (Neisser, 2021). In addition to the conventional analysis of organics using SIFT‐MS (Smith et al., 2020), analysis of ammonia (Smith et al., 2015) and the acid vapors (Ghislain et al., 2019; Hera et al., 2017) has been demonstrated. Coupled with the ability to analyze air continuously 24 h a day and at high sensitivity (Lehnert, Behrendt, et al., 2020; Prince et al., 2010), SIFT‐MS instrumentation utilizing nitrogen carrier gas provides comprehensive analysis of VOCs and inorganic gases, protecting product, manufacturing equipment, and production staff.

## 7 FOOD‐FLAVOR, PHARMACEUTICALS, AND MATERIAL EMISSIONS ANALYSIS

Commodities for human consumption or application, consumer products, and pharmaceutical application areas are combined in one section. VOC emissions from materials, including packaging, are important in both areas. Material emissions are, of course, a wider issue and contribute to indoor air quality. Natural or mechanical ventilation also impact outdoor air quality in urban areas.

### 7.1 Food flavor, spoilage, and authentication

Food analysis has received considerable attention from SIFT‐MS because VOCs contribute to aroma, with both good and bad effects. In mid‐2019, a comprehensive review was published on this area (Langford et al., 2019), and complementary overviews on industry‐focused food‐flavor applications appeared in late 2021 (Barringer, 2021; Padayachee & Langford, 2021). In the last 3 years, SIFT‐MS was applied to the area of food spoilage: in packaged leaf salads (Dryahina et al., 2020), shredded carrot (Kuuliala et al., 2021), and packaged salmon (Dalsvåg et al., 2022; Kuuliala et al., 2019). It is currently very active in a range of food product quality application, including packaged Iberian hams (Geeraerts et al., 2019), strawberry ripeness (Vendel et al., 2019), screening of commercial dairy products (Wood et al., 2021), authentication of extra‐virgin Argan oils (Kharbach et al., 2018; Kharbach et al., 2022), authentication of adulteration of extra virgin olive oil (Ozcan‐Sinir, 2020), classification of wine grapes by variety at various stages of ripening (Baerenzung dit Baron et al., 2022) and authentication of the origin of ewe cheeses (Reyrolle et al., 2022).

Following the wide range of SIFT‐MS analyses of tomatoes, tomatillos, strawberries, jalapeno peppers, bell peppers, garlic, cocoa beans and liquors, cashew nuts, almonds, pumpkin seeds, and carrots (Sumonsiri & A Barringer, 2013), the research group at the Ohio State University has continued research on diverse food chemistry applications of SIFT‐MS, with studies on garlic oil encapsulation (Yu et al., 2019), deodorization of garlic‐derived volatile organosulfur compounds (Castada & Barringer, 2019), differences between garlic varieties (Ozcan‐Sinir & Barringer, 2020) and thermal degradation of *p*‐hydroxybenzoic acid in various oils, including macadamia nut oil (Castada et al., 2020).

### 7.2 Pharmaceutical quality and contamination

Few pharmaceutical products are volatile (though some may be delivered in the form of aerosols). Nevertheless, VOCs find application as solvents in manufacture or purification and they may also be formed as byproducts in synthesis or through degradation during storage, and may migrate into pharmaceutical products from packaging. GC‐based techniques are most utilized for these analyses in the pharmaceutical industry. For a new technique to be adopted by the pharmaceutical industry, it is essential that analytical methods be validated according to industry guidelines, such as (ICH, 2005). The feasibility of a SIFT‐MS validation method has been demonstrated recently using a model aqueous headspace system (Perkins & Langford, 2021b) that provides a platform from which to develop fully validated applications. For routine analysis, SIFT‐MS has potential economic benefits through increased throughput and simplification of sample preparation (compared to GC‐MS analysis of chromatographically challenging compounds), but future in‐process and continuous manufacturing applications are also probable (S. L. Lee et al., 2015).

The most widely employed procedure for analysis of volatiles residual solvents places them into classes (USP, 2019a). Class 1 solvents have unacceptable toxicity (or, for 1,1,1‐trichloroethane, ozone depletion potential) and should be avoided whenever possible, Class 2 solvents have less severe toxicity and should be limited to protect patients, while Class 3 solvents, with their low toxic potential, should be used where practical. As promulgated, it is a method that utilizes headspace‐GC (with flame ionization detection, FID, or MS detection), but alternative procedures can also be validated (USP, 2019b). Recently, a feasibility study was conducted across Class 1 and 2 solvents for water‐soluble drug products using headspace SIFT‐MS analysis, with full validation of the Class 2A and 2B solvents according to USP〈1467〉 (Biba et al., 2021). This validation study met USP〈1467〉 acceptance criteria for all solvents except pyridine and hexane, for which recoveries failed in selected drug products. Future work will seek to address these issues, while also (i) broadening validation of the alternative procedure to cover Class 1, (ii) investigating different sample preparation approaches for the Class 2C solvents (with poor headspace partitioning that makes static headspace analysis a challenge for an analytical technique), and (iii) evaluating options for SIFT‐MS analysis of drug products that are insoluble in water.

SIFT‐MS also has potential to target volatile impurities. Formaldehyde analysis in drug delivery devices was validated successfully for regulatory submission. Direct‐injection SIFT‐MS has greatly simplified sample preparation and analysis compared to the traditional approach, which comprises 2,4‐dinitrophenylhydrazine (DNPH) derivatization, followed by solvent elution, then analysis using high‐performance liquid chromatography with ultraviolet detection (HPLC‐UV). In 2018, volatile, carcinogenic nitrosamines were discovered in sartan‐type drug products that are widely used to regulate blood pressure resulting in product recalls. They have since been found in other drug products, but all sources of nitrosamines are yet to be identified. (See WHO Information Note “Update on nitrosamine impurities” https://cdn.who.int/media/docs/default-source/essential-medicines/medical-alert-2019/informationnotenitrosamine-impurities-nov2019en.pdf and the notes from the Office of New Drugs, United States Food and Drug Administration Health Risk Assessment and Mitigation Public Workshop “Nitrosamines as Impurities in Drugs,” March 29–30, 2021. https://www.fda.gov/media/150932/download.) Discussions about the extent of drug product testing that are required, and the high‐sensitivity analytical methods to do so, are ongoing. However, it is known that nitrosamines are readily detected using SIFT‐MS (Langford et al., 2015).

### 7.3 Material VOC emissions and packaging

Synthetic and natural materials used in many modern consumer and commercial products from packaging to components used in motor vehicle interiors (see Section 6.1) emit measurable levels of VOCs. These VOCs vary from the innocuous to very harmful, and those that present a health risk must be controlled. SIFT‐MS is ideally suited to detecting and quantifying these volatiles. To date, polymeric materials have received the most attention. Quantitation of residual monomers in polymer headspace is straightforward and can be achieved at throughputs of 12 samples/hour using static real‐time headspace analysis. Furthermore, the ability of SIFT‐MS to directly analyze high‐polarity VOCs such as formaldehyde and acetaldehyde (Smith et al., 2014) greatly simplifies analysis compared to GC or LC, where derivatization is required to achieve effective chromatography.

Quantitative determination of residual volatiles in the polymer relies upon stripping of all volatiles from the sample using, for example, dynamic headspace analysis. Because exhaustive extraction is ordinarily a very long procedure, the multiple headspace extraction (MHE) technique was developed (Kolb & Ettre, 2006) to achieve the same result by using extrapolation of a limited number of headspace measurements that follow headspace purge and regeneration cycles. Recently, a detailed MHE protocol has been published for SIFT‐MS (Perkins & Langford, 2022) using styrene analysis from polystyrene as the prototypical system. Relative standard deviations across six replicate samples were 1.4%. Earlier work had demonstrated that formaldehyde residue is readily quantified in polyoxymethylene (POM) polymer. Due to multiple reanalysis of the same sample, MHE is a very protracted procedure when coupled with GC because of the long run times (tens of minutes, which is the rate‐limiting step). In contrast, headspace analysis using SIFT‐MS takes 2 min, so one sample can be analyzed while the headspace of up to 11 other samples is generated. The number of samples that can be analyzed in parallel is then dependent on the time taken for the headspace analyze‐and‐purge cycle relative to the time to reach equilibrium. For polystyrene, an eightfold throughput enhancement was achieved with SIFT‐MS compared to the equivalent GC‐MS method.

Targeted static headspace and MHE methods are well suited to determination of known volatile residues (especially in virgin polymer), but recycled plastics have varied usage histories and therefore have a substantially higher risk of releasing other, nontargeted compounds. With high probability of significant inter‐sample variation, an untargeted static headspace approach is preferable. Nonconforming samples are detected quickly by utilizing rapid full‐scan SIFT‐MS analysis coupled with multivariate statistical data processing. The feasibility of this approach has been demonstrated recently for virgin high‐density polyethylene (HDPE) pellets and recycled HDPE (Langford & Perkins, 2022). The rapid switching of reagent ions in SIFT‐MS instruments enabled acquisition of full‐scan spectra with all three SIFT‐MS reagent cations in one 40‐s analysis (i.e., during a single headspace injection). All samples were successfully distinguished, demonstrating that untargeted SIFT‐MS analysis coupled with multivariate statistical analysis can be applied to rapid differentiation of recycled HDPE, for example, to assure pellet volatile emissions conform to the standard required for their intended use.

Besides safety concerns, packaging materials in contact with foods, pharmaceuticals, and other consumer products can transmit volatiles to these products that have sensory impact. This can negatively influence consumer acceptance. However, human sensory testing is expensive (due to the use of multiple highly‐trained panelists) and has low daily sample throughput. To address these limitations, SIFT‐MS has been evaluated for its ability to provide high‐throughput instrument‐based prediction of odor intensity rating and odor note for paper materials used in packaging (Langford et al., 2021). This study concluded that SIFT‐MS has potential for sensory classification of paper samples, both for odor intensity ratings and identification of sensory odors.

**Figure 6:**
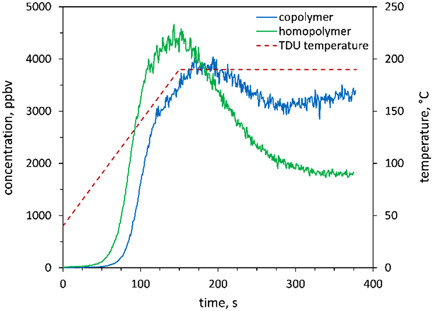
Real‐time monitoring by SIFT‐MS of thermal extraction of formaldehyde from single copolymer and homopolymer granules. The temperature ramp (60°C min^−1^) and hold (at 190°C) of the thermal desorption unit, TDU, is shown on the right‐hand axis. SIFT‐MS, selected ion flow tube mass spectrometry. [Color figure can be viewed at wileyonlinelibrary.com]

SIFT‐MS can provide unique insights into materials by providing real‐time monitoring of volatiles as they are thermally extracted using integrated thermal desorption‐SIFT‐MS (Langford et al., 2021). Figure 6 shows the formaldehyde release profiles from single granules of POM copolymer and homopolymer manufactured for injection molding applications. When holding samples near the recommended molding temperature (190°C), both polymers emit significant concentrations of formaldehyde throughout the analysis. The hold temperature is above the melting point for both forms of polymer (160–180°C), so these emissions arise from the molten polymer rather than an intact polymer granule. Thermal extraction of POM is greatly simplified compared to conventional chromatographic approaches due to the derivatization‐free, direct analysis that is provided by SIFT‐MS.

Novel material emissions applications of SIFT‐MS have been uncovered in heritage science. Frequently in parallel with GC‐MS or pyrolysis‐GC‐MS for validation purposes, SIFT‐MS has been utilized for noninvasive identification of volatiles and subsequent “spectral fingerprinting.” Heritage applications to date include analysis of volatiles emitted from comic books preserved in plastic packaging (La Nasa, Mattonai, et al., 2019), natural and synthetic paint varnishes (La Nasa, Modugno, et al., 2019), organic residues from ancient Egyptian archaeological finds (La Nasa et al., 2020), and in‐tact unique ancient Egyptian artefacts (La Nasa et al., 2022). Significantly, although some of the materials studied are clearly being analyzed outside the SIFT‐MS instrument's concentration linear range, qualitative analysis proceeds successfully, volatiles are identified, and the pattern recognition approach effectively classifies the materials.

## 8 CONCLUDING REMARKS AND FUTURE DIRECTIONS

Since the inception of SIFT‐MS, which initially took the form of a large and heavy strictly laboratory instrument, it quickly became clear that, potentially, this is a tool that could be used for real‐time analysis of humid air in many environments. And so, it has turned out, as the information in this review demonstrates. In the interim, major developments have been accomplished in the instrumentation culminating in the much smaller *Profile 3* and the eminently successful *Voice200* series of SIFT‐MS. These developments have been described in previous publications (Dummer et al., 2013; Španěl & Smith, 2011; Zhu et al., 2017) and need not be repeated in this review. Worthy of note are the subtle yet important modifications of heating of the carrier gas and the major step to include reagent anions with reagent cations, which has broadened the range of volatile compounds that can be analyzed to include those that cannot be analyzed using reagent cations. Further to these additions, the use of nitrogen carrier gas to replace scarce helium has been demonstrated to be a valid option for long‐time monitoring of toxic trace gases in industrial and environmental scenarios, which is advantageous in costs and supply.

Underpinning SIFT‐MS analysis is a proper understanding of the analytical gas‐phase ion chemistry and the building of an extensive kinetics library that can provide identification and quantification of large numbers of trace VOCs that coexist in a medium (ambient air, very humid exhaled breath, and other atmospheres), either in real‐time at pptv concentrations obviating sample collection or by controlled sampling and collection for off‐line analysis by SIFT‐MS. The kinetics library is continuously being extended as more compounds are detected resulting from the broader widespread use of SIFT‐MS, which is increasingly becoming adopted in industry, for basic research and as a mainstream analytical tool for routine analysis.

There are few situations where trace gas analysis is needed that cannot benefit from SIFT‐MS. SIFT‐MS is not well suited for compounds with low volatility, poor partitioning to the vapor phase, or compounds that do not react with the reagent ions in any useful way (e.g., carbon monoxide and hydrogen). Also, complex systems (e.g., biological) where there are many potential isomeric and isobaric species need separation techniques. Yet there are still ways by which the analytical procedure and instrumentation can be improved and developed. Again, a better understanding of ion chemistry will always provide insights and benefits. An obvious point for further investigation is the use of nitrogen carrier gas, which has complications above those of helium, as indicated in a section of this review. Further, it seems likely that other reagent cations and anions will be discovered, as shown by the SIFT work on NH_4_^+^ reagent ions. New reagents will probably be able to analyze only selected compounds, but they could be more selective and less ambiguous analytically. Some of the research and industrial applications described in this review have only been touched upon, such as emissions from solids (drugs) in the pharmaceutical sector, but the real‐time rapid analysis offered by SIFT‐MS will be advantageous as its unique and versatile features become more readily recognized. There is a greater analytical reach of SIFT‐MS if the trace gases in a sample can be extracted and stored without degradation and quantified by SIFT‐MS off‐line. Such is the research into thermal adsorption/desorption that is being trialed in some clinical laboratories for breath collection and analysis. In this way, the exhaled breath of large numbers of patients from remote clinical sites could be accessed, which is considered necessary to account for biological variations in human beings. The temporal thermal extraction of volatiles from solids such as polymers and other compounds in material science studies is another area that could benefit from the real time aspect of SIFT‐MS. New opportunities are bound to open in routine laboratory testing in various areas, e.g. hydrogen fuel or cosmetics products.

## References

[mas21835-bib-0001] Adams NG , Smith D. Selected ion flow tube (SIFT)—technique for studying ion‐neutral reactions. Int. J. Mass Spectrom. Ion Process. 1976;21:349‐359. 10.1016/0020-7381(76)80133-7

[mas21835-bib-0002] Adams NG , Smith D , Paulson JF. An experimental survey of the reactions of NH_n_ ^+^ ions (n = 0 to 4) with several diatomic and polyatomic molecules at 300 K. J. Chem. Phys. 1980;72:288‐297. 10.1063/1.438893

[mas21835-bib-0003] Ajibola OA , Smith D , Španěl P , Ferns GAA. Effects of dietary nutrients on volatile breath metabolites. J. Nutritional Sci. 2013;2:e34. 10.1017/jns.2013.26 PMC4153095 25191584

[mas21835-bib-0004] Allen ND , Perkins M , Bacquart T , Li J. Determining modified reaction parameters for the real‐time measurement of BTEX in biogas and nitrogen using selected ion flow tube mass spectrometry (SIFT‐MS). Accreditation Qual. Assur. 2019;24:361‐368. 10.1007/s00769-019-01394-8

[mas21835-bib-0005] Allpress C , Crittenden D , Ma J , McEwan M , Robinson S , Wilson P , Wu M. Real‐time differentiation of ethylbenzene and the xylenes using selected ion flow tube mass spectrometry. Rapid Commun. Mass Spectrom. 2019;33:1844‐1849. 10.1002/rcm.8550 31411756

[mas21835-bib-0006] Baerenzung dit Baron T , Yobrégat O , Jacques A , Simon V , Geffroy O . A novel approach to discriminate the volatilome of Vitis vinifera berries by selected ion flow tube mass spectrometry analysis and chemometrics. Food Res. Int. 2022;157:111434. 10.1016/j.foodres.2022.111434 35761674

[mas21835-bib-0007] Barringer S. From mold worms to fake honey: using SIFT‐MS to improve food quality. In: Beauchamp JD, ed. Dynamic Flavor: Capturing Aroma Using Real‐Time Mass Spectrometry. American Chemical Society; 2021:99‐105. 10.1021/bk-2021-1402.ch008

[mas21835-bib-0008] Baur X , Poschadel B , Budnik LT. High frequency of fumigants and other toxic gases in imported freight containers—an underestimated occupational and community health risk. Occup. Environ. Med. 2010;67:207‐212. 10.1136/oem.2008.043893 19858536

[mas21835-bib-0009] Beauchamp J , Davis C , Pleil J. 2020. Breathborne Biomarkers and the Human Volatilome: Elsevier Science. 10.1016/C2018-0-04980-4

[mas21835-bib-0010] Belluomo I , Boshier PR , Myridakis A , Vadhwana B , Markar SR , Spanel P , Hanna GB. Selected ion flow tube mass spectrometry for targeted analysis of volatile organic compounds in human breath. Nat. Protoc. 2021;16:3419‐3438. 10.1038/s41596-021-00542-0 34089020

[mas21835-bib-0011] Biba E , Perkins M , Langford V. Stimuli to the revision process: high‐throughput residual solvent analysis using selected ion flow tube mass spectrometry (SIFT‐MS). United States Pharmacopeia. Pharmacopeial Forum 2021;47:1.

[mas21835-bib-0012] Bierbaum VM. Go with the flow: fifty years of innovation and ion chemistry using the flowing afterglow. Int. J. Mass Spectrom. 2015;377:456‐466. 10.1016/j.ijms.2014.07.021

[mas21835-bib-0013] Borras E , Tortajada‐Genaro LA , Rodenas M , Vera T , Speak T , Seakins P , Shaw MD , Lewis AC , Munoz A. On‐line solid phase microextraction derivatization for the sensitive determination of multi‐oxygenated volatile compounds in air. Atmos. Meas. Tech. 2021;14:4989‐4999. 10.5194/amt-14-4989-2021

[mas21835-bib-0014] Boshier PR , Cushnir JR , Mistry V , Knaggs A , Španěl P , Smith D , Hanna GB. On‐line, real time monitoring of exhaled trace gases by SIFT‐MS in the perioperative setting: a feasibility study. Analyst 2011;136:3233‐3237. 10.1039/c1an15356k 21717028

[mas21835-bib-0015] Bouchoux G , Salpin JY , Leblanc D. A relationship between the kinetics and thermochemistry of proton transfer reactions in the gas phase. Int. J. Mass Spectrom. Ion Process. 1996;153:37‐48. 10.1016/0168-1176(95)04353-5

[mas21835-bib-0016] Bruneel J , Follert JLH , Laforce B , Vincze L , Van Langenhove H , Walgraeve C. Dynamic performance of a fungal biofilter packed with perlite for the abatement of hexane polluted gas streams using SIFT‐MS and packing characterization with advanced X‐ray spectroscopy. Chemosphere 2020;253:126684. 10.1016/j.chemosphere.2020.126684 32464772

[mas21835-bib-0017] Bruneel J , Walgraeve C , Dumortier S , Stockman J , Demeyer P , Van Langenhove H. Increasing mass transfer of volatile organic compounds in air scrubbers: a fundamental study for different gas‐liquid systems. J. Chem. Technol. Biotechnol. 2018;93:1468‐1476. 10.1002/jctb.5515

[mas21835-bib-0018] Bruneel J , Walgraeve C , Mukurarinda J , Boon N , Van Langenhove H. Biofiltration of hexane, acetone and dimethyl sulphide using wood, compost and silicone foam. J. Chem. Technol. Biotechnol. 2018;93:2234‐2243. 10.1002/jctb.5566

[mas21835-bib-0019] Bruneel J , Walgraeve C , Van Huffel K , Van Langenhove H. Determination of the gas‐to‐liquid partitioning coefficients using a new dynamic absorption method (DynAb method). Chem. Eng. J. 2016;283:544‐552. 10.1016/j.cej.2015.07.053

[mas21835-bib-0020] Bryant DJ , Dixon WJ , Hopkins JR , Dunmore RE , Pereira K , Shaw M , Squires FA , Bannan TJ , Mehra A , Worrall SD , Bacak A , Coe H , Percival CJ , Whalley LK , Heard DE , Slater EJ , Ouyang B , Cui TQ , Surratt JD , Liu D , Shi ZB , Harrison R , Sun YL , Xu WQ , Lewis AC , Lee JD , Rickard AR , Hamilton JF. Strong anthropogenic control of secondary organic aerosol formation from isoprene in Beijing. Atmos. Chem. Phys. 2020;20:7531‐7552. 10.5194/acp-20-7531-2020

[mas21835-bib-0021] Carroll W , Lenney W , Wang TS , Španěl P , Alcock A , Smith D. Detection of volatile compounds emitted by Pseudomonas aeruginosa using selected ion flow tube mass spectrometry. Pediatr. Pulmonol. 2005;39:452‐456. 10.1002/ppul.20170 15765542

[mas21835-bib-0022] Castada HZ , Barringer SA. Online, real‐time, and direct use of SIFT‐MS to measure garlic breath deodorization: a review. Flavour Fragr. J. 2019;34:299‐306. 10.1002/ffj.3503

[mas21835-bib-0023] Castada HZ , Sun ZY , Barringer SA , Huang XS. Thermal degradation of p‐hydroxybenzoic acid in macadamia nut oil, olive oil, and corn oil. J. Am. Oil Chem. Soc. 2020;97:289‐300. 10.1002/aocs.12331

[mas21835-bib-0024] Castelvetro V , Corti A , Biale G , Ceccarini A , Degano I , La Nasa J , Lomonaco T , Manariti A , Manco E , Modugno F , Vinciguerra V. New methodologies for the detection, identification, and quantification of microplastics and their environmental degradation by‐products. Environ. Sci. Pollut. Res. 2021;28:46764‐46780. 10.1007/s11356-021-12466-z PMC8384832 33502712

[mas21835-bib-0025] Chippendale TWE , Gilchrist FJ , Španěl P , Alcock A , Lenney W , Smith D. Quantification by SIFT‐MS of volatile compounds emitted by Aspergillus fumigatus cultures and in co‐culture with Pseudomonas aeruginosa, Staphylococcus aureus and Streptococcus pneumoniae. Anal. Methods 2014a;6:8154‐8164. 10.1039/C4ay01217h

[mas21835-bib-0026] Chippendale TWE , Gilchrist FJ , Španěl P , Alcock A , Lenney W , Smith D. Quantification by SIFT‐MS of volatile compounds emitted by in vitro cultures of S. aureus, S. pneumoniae, and H. influenzae isolated from patients with respiratory diseases. Anal. Methods 2014b;6:2460‐2472. 10.1039/C4ay00209a

[mas21835-bib-0027] Chippendale TWE , Španěl P , Smith D. Time‐resolved selected ion flow tube mass spectrometric quantification of the volatile compounds generated by E. coli JM109 cultured in two different media. Rapid Commun. Mass Spectrom. 2011;25:2163‐2172. 10.1002/rcm.5099 21710596

[mas21835-bib-0028] Civiš M , Civiš S , Sovová K , Dryahina K , Španěl P , Kyncl M. Laser ablation of FOX‐7: proposed mechanism of decomposition. Anal. Chem. 2011;83:1069‐1077. 10.1021/ac1028769 21226460

[mas21835-bib-0029] Civis S , Civis M , Sovova K , Dryahina K , Kubista J , Skrehot P , Španěl P , Kyncl M. Selected ion flow tube mass spectrometry analyses of laser decomposition products of a range of explosives and ballistic propellants. Anal. Methods 2016;8:1145‐1150. 10.1039/c5ay03039k

[mas21835-bib-0030] Crilley LR , Kramer LJ , Ouyang B , Duan J , Zhang WQ , Tong SR , Ge MF , Tang K , Qin M , Xe PH , Shaw M , Lewis AC , Mehra A , Bannan TJ , Worrall SD , Priestley M , Bacak A , Coe H , Allan J , Percival CJ , Popoola OAM , Jones RL , Bloss WJ. Intercomparison of nitrous acid (HONO) measurement techniques in a megacity (Beijing). Atmos. Meas. Tech. 2019;12:6449‐6463. 10.5194/amt-12-6449-2019

[mas21835-bib-0031] Custer TG , Kato S , Fall R , Bierbaum VM. Negative‐ion CIMS: analysis of volatile leaf wound compounds including HCN. Int. J. Mass Spectrom. 2003;223‐224:427‐446. 10.1016/S1387-3806(02)00930-2

[mas21835-bib-0032] Dalsvåg H , Cropotova J , Jambrak AR , Janči T , Španěl P , Dryahina K , Smith D , Rustad T. Mass spectrometric quantification of volatile compounds released by fresh Atlantic salmon stored at 4°C under modified atmosphere packaging and vacuum packaging for up to 16 days. ACS Food Sci. Technol. 2022;2:400‐414. 10.1021/acsfoodscitech.1c00259

[mas21835-bib-0033] Davies SJ , Španěl P , Smith D. Breath analysis of ammonia, volatile organic compounds and deuterated water vapor in chronic kidney disease and during dialysis. Bioanalysis 2014;6:843‐857. 24702114 10.4155/bio.14.26

[mas21835-bib-0034] Den W , Hu SC , Garza CM , Zargar OA. Review‐airborne molecular contamination: recent developments in the understanding and minimization for advanced semiconductor device manufacturing. Ecs J. Solid State Sci. Technol. 2020;9:064003. 10.1149/2162-8777/aba080

[mas21835-bib-0035] DePuy C , Bierbaum V , Flippin L , Grabowski J , King G , Schmitt R , Sullivan S. Gas‐phase reactions of anions with substituted silanes. J. Am. Chem. Soc. 1980;102:5012‐5015.

[mas21835-bib-0036] DePuy CH , Bierbaum VM. 1987. Proton Transfer Reactions of Anions. In: Ausloos P , Lias SG , Editors. Structure/Reactivity and Thermochemistry of Ions. Dordrecht: Springer Netherlands. p 293‐303. 10.1007/978-94-009-3787-1_15

[mas21835-bib-0037] DePuy CH , Grabowski JJ , Bierbaum VM. Chemical reactions of anions in the gas‐phase. Science 1982;218:955‐960. 10.1126/science.218.4576.955 17790572

[mas21835-bib-0038] Doepke A , Streicher RP. Source apportionment and quantification of liquid and headspace leaks from closed system drug‐transfer devices via selected ion flow tube mass spectrometry (SIFT‐MS). Plos One 2021;16:e0258425. 10.1371/journal.pone.0258425 34735484 PMC8568112

[mas21835-bib-0039] Dryahina K , Pospisilova V , Sovova K , Shestivska V , Kubista J , Spesyvyi A , Pehal F , Turzikova J , Votruba J , Španěl P. Exhaled breath concentrations of acetic acid vapour in gastro‐esophageal reflux disease. J. Breath Res. 2014;8:037109. 10.1088/1752-7155/8/3/037109 25189108

[mas21835-bib-0040] Dryahina K , Smith D , Bortlik M , Machkova N , Lukas M , Španěl P. Pentane and other volatile organic compounds, including carboxylic acids, in the exhaled breath of patients with Crohn's disease and ulcerative colitis. J. Breath Res. 2018;12:016002. 10.1088/1752-7163/aa8468 28781264

[mas21835-bib-0041] Dryahina K , Smith D , Španěl P. Quantification of methane in humid air and exhaled breath using selected ion flow tube mass spectrometry. Rapid Commun. Mass Spectrom. 2010;24:1296‐1304. 10.1002/rcm.4513 20391601

[mas21835-bib-0042] Dryahina K , Smith D , Španěl P. Quantification of volatile compounds released by roasted coffee by selected ion flow tube mass spectrometry. Rapid Commun. Mass Spectrom. 2018;32:739‐750. 10.1002/rcm.8095 29486530

[mas21835-bib-0043] Dryahina K , Som S , Smith D , Španěl P. Characterization of spoilage‐related volatile organic compounds in packaged leaf salads. Flavour Fragr. J. 2020;35:24‐33. 10.1002/ffj.3535

[mas21835-bib-0044] Dryahina K , Španěl P , Pospisilova V , Sovova K , Hrdlicka L , Machkova N , Lukas M , Smith D. Quantification of pentane in exhaled breath, a potential biomarker of bowel disease, using selected ion flow tube mass spectrometry. Rapid Commun. Mass Spectrom. 2013;27:1983‐1992. 10.1002/rcm.6660 23939966

[mas21835-bib-0045] Dummer J , Storer M , Sturney S , Scott‐Thomas A , Chambers S , Swanney M , Epton M. Quantification of hydrogen cyanide (HCN) in breath using selected ion flow tube mass spectrometry‐HCN is not a biomarker of Pseudomonas in chronic suppurative lung disease. J. Breath Res. 2013;7:017105. 10.1088/1752-7155/7/1/017105 23445778

[mas21835-bib-0046] EPA . 1999. Compendium of Methods for the Determination of Toxic Organic Compounds in Ambient Air. United States Environmental Protection Agency Ohio.

[mas21835-bib-0047] Francis GJ , Milligan DB , McEwan MJ. Detection and quantification of chemical warfare agent precursors and surrogates by selected ion flow tube mass spectrometry. Anal. Chem. 2009;81:8892‐8899. 10.1021/ac901486c 19788274

[mas21835-bib-0048] Geeraerts W , Borremans W , De Vuyst L , Leroy F , Van Kerrebroeck S. The application of selected ion flow tube‐mass spectrometry to follow volatile formation in modified‐atmosphere‐packaged cooked ham. Food Res. Int. 2019;123:601‐611. 10.1016/j.foodres.2019.05.035 31285009

[mas21835-bib-0049] Ghislain M , Costarramone N , Sotiropoulos JM , Pigot T , Van Den Berg R , Lacombe S , Le Bechec M. Direct analysis of aldehydes and carboxylic acids in the gas phase by negative ionization selected ion flow tube mass spectrometry: quantification and modelling of ion‐molecule reactions. Rapid Commun. Mass Spectrom. 2019;33:1623‐1634. 10.1002/rcm.8504 31216077

[mas21835-bib-0050] Ghislain M , Reyrolle M , Sotiropoulos JM , Pigot T , Plaisance H , Le Bechec M. Study of the chemical ionization of organophosphate esters in air using selected ion flow tube‐mass spectrometry for direct analysis. J. Am. Soc. Mass Spectrom. 2022;33:865‐874. 10.1021/jasms.2c00060 35416666

[mas21835-bib-0051] Gilchrist FJ , Alcock A , Belcher J , Brady M , Jones A , Smith D , Španěl P , Webb K , Lenney W. Variation in hydrogen cyanide production between different strains of Pseudomonas aeruginosa. Eur. Respir. J. 2011;38:409‐414. 10.1183/09031936.00166510 21273393

[mas21835-bib-0052] Gilchrist FJ , Belcher J , Jones AM , Smith D , Smyth AR , Southern KW , Španěl P , Webb AK , Lenney W. Exhaled breath hydrogen cyanide as a marker of early Pseudomonas aeruginosa infection in children with cystic fibrosis. ERJ Open Res. 2015;1:00044‐02015. 10.1183/23120541.00044-2015 27730156 PMC5005121

[mas21835-bib-0053] Gilchrist FJ , Bright‐Thomas RJ , Jones AM , Smith D , Španěl P , Webb AK , Lenney W. Hydrogen cyanide concentrations in the breath of adult cystic fibrosis patients with and without Pseudomonas aeruginosa infection. J. Breath Res. 2013;7:026010. 10.1088/1752-7155/7/2/026010 23680696

[mas21835-bib-0054] Gilchrist FJ , Sims H , Alcock A , Belcher J , Jones AM , Smith D , Španěl P , Webb AK , Lenney W. Quantification of hydrogen cyanide and 2‐aminoacetophenone in the headspace of Pseudomonas aeruginosa cultured under biofilm and planktonic conditions. Anal. Methods 2012;4:3661‐3665. 10.1039/c2ay25652e

[mas21835-bib-0055] Gilchrist FJ , Španěl P , Smith D , Lenney W. The in vitro identification and quantification of volatile biomarkers released by cystic fibrosis pathogens. Anal. Methods 2015;7:818‐824. 10.1039/c4ay02981j

[mas21835-bib-0056] Haick H , Broza YY , Mochalski P , Ruzsanyi V , Amann A. Assessment, origin, and implementation of breath volatile cancer markers. Chem. Soc. Rev. 2014;43:1423‐1449. 10.1039/c3cs60329f 24305596 PMC4909138

[mas21835-bib-0057] Hastie C , Thompson A , Perkins M , Langford VS , Eddleston M , Homer NZM. Selected ion flow tube‐mass spectrometry (SIFT‐MS) as an alternative to gas chromatography/mass spectrometry (GC/MS) for the analysis of cyclohexanone and cyclohexanol in plasma. Acs Omega 2021;6:32818‐32822. 10.1021/acsomega.1c03827 34901631 PMC8655936

[mas21835-bib-0058] Hegen O , Gomez JIS , Schlogl R , Ruland H. The potential of NO^+^ and O_2_ ^+•^ in switchable reagent ion proton transfer reaction time‐of‐flight mass spectrometry. Mass Spectrom. Rev. 2022:e21770. 10.1002/mas.21770 35076949

[mas21835-bib-0059] Hera D , Langford VS , McEwan MJ , McKellar TI , Milligan DB. Negative reagent ions for real time detection using SIFT‐MS. Environments 2017;4:16. 10.3390/environments4010016

[mas21835-bib-0060] Hermabessiere L , Himber C , Boricaud B , Kazour M , Amara R , Cassone A‐L , Laurentie M , Paul‐Pont I , Soudant P , Dehaut A , Duflos G. Optimization, performance, and application of a pyrolysis‐GC/MS method for the identification of microplastics. Anal. Bioanal. Chem. 2018;410:6663‐6676. 10.1007/s00216-018-1279-0 30051208

[mas21835-bib-0061] Heynderickx PM , Španěl P , Van Langenhove H. Quantification of octanol‐water partition coefficients of several aldehydes in a bubble column using selected ion flow tube mass spectrometry. Fluid Phase Equilibria 2014;367:22‐28. 10.1016/j.fluid.2014.01.017

[mas21835-bib-0062] Heynderickx PM , Van Huffel K , Dewulf J , Van Langenhove H. 2012. SIFT‐MS for livestock emission characterization: application of similarity coefficients. In: DelRosso R, Pierucci S, Klemes JJ, Editors. Nose 2012: 3rd International Conference on Environmental Odour Monitoring and Control. p 157‐162. 10.3303/cet1230027

[mas21835-bib-0063] Heynderickx PM , Van Huffel K , Dewulf J , Van Langenhove H. Application of similarity coefficients to SIFT‐MS data for livestock emission characterization. Biosyst. Eng. 2013;114:44‐54. 10.1016/j.biosystemseng.2012.10.004

[mas21835-bib-0064] Hien TT , Huy DH , Dominutti PA , Chi NDT , Hopkins JR , Shaw M , Forster G , Mills G , Le HA , Oram D. Comprehensive volatile organic compound measurements and their implications for ground‐level ozone formation in the two main urban areas of Vietnam. Atmos. Environ. 2022;269:118872. 10.1016/j.atmosenv.2021.118872

[mas21835-bib-0065] Hinz R , 't Mannetje A , Glass B , McLean D , Douwes J. Airborne fumigants and residual chemicals in shipping containers arriving in New Zealand. Ann. Work Expo. Health 2022;66:481‐494. 10.1093/annweh/wxab090 34657959 PMC9030136

[mas21835-bib-0066] Hinz R , 't Mannetje A , Glass B , McLean D , Pearce N , Douwes J. Exposures to fumigants and residual chemicals in workers handling Cargo from shipping containers and export logs in New Zealand. Ann. Work Expo. Health 2020;64:826‐837. 10.1093/annweh/wxaa052 32504467

[mas21835-bib-0067] Hryniuk A , Ross BM. Detection of acetone and isoprene in human breath using a combination of thermal desorption and selected ion flow tube mass spectrometry. Int. J. Mass Spectrom. 2009;285:26‐30. 10.1016/j.ijms.2009.02.027

[mas21835-bib-0068] Hwang K , An JG , Lee S , Choi W , Yim UH. A study on the ozone formation potential of volatile organic compounds in Busan using SIFT‐MS. J. Korean Soc. Atmos. Environ. 2020;36:645‐668. 10.5572/Kosae.2020.36.5.645

[mas21835-bib-0069] ICH . 2005. International Council for Harmonization of Technical Requirements for Pharmaceuticals for Human Use guideline: Validation of analytical procedures: text and methodology Q2 (R1).

[mas21835-bib-0070] Kaus C , Thomas B , Breuer D. Application of SIFT‐MS for selective real‐time online monitoring of dynamic test gas atmospheres and measurement of canister‐collected whole air samples. Gefahrst Reinhalt L. 2022;82:67‐73. 10.1002/rcm.4574

[mas21835-bib-0071] Keer S , Taptiklis P , Glass B , McLean D , McGlothlin JD , Douwes J. Determinants of airborne solvent exposure in the collision repair industry. Ann. Work Expos. Health 2018;62:871‐883. 10.1093/annweh/wxy047 29912331

[mas21835-bib-0072] Kharbach M , Kamal R , Mansouri MA , Marmouzi I , Viaene J , Cherrah Y , Alaoui K , Vercammen J , Bouklouze A , Vander Heyden Y. Selected‐ion flow‐tube mass‐spectrometry (SIFT‐MS) fingerprinting versus chemical profiling for geographic traceability of Moroccan Argan oils. Food Chem. 2018;263:8‐17. 10.1016/j.foodchem.2018.04.059 29784331

[mas21835-bib-0073] Kharbach M , Yu HW , Kamal R , Marmouzi I , Alaoui K , Vercammen J , Bouklouze A , Vander Heyden Y. Authentication of extra virgin Argan oil by selected‐ion flow‐tube mass‐spectrometry fingerprinting and chemometrics. Food Chem. 2022;383:132565. 10.1016/j.foodchem.2022.132565 35245834

[mas21835-bib-0074] Kim KJ , Kim HJ , Son D , Jeong NR , Yun HG , Han SW , You S , Kim C‐J , Lee SH. Identification of plant response to the human behavior of crushing plants. J. People Plants Environ. 2019;22:593‐600.

[mas21835-bib-0075] Knizek A , Dryahina K , Španěl P , Kubelik P , Kavan L , Zukalova M , Ferus M , Civis S. Comparative SIFT‐MS, GC‐MS and FTIR analysis of methane fuel produced in biogas stations and in artificial photosynthesis over acidic anatase TiO_2_ and montmorillonite. J. Mol. Spectr. 2018;348:152‐160. 10.1016/j.jms.2017.10.002

[mas21835-bib-0076] Kolb B , Ettre LS. 2006. Static Headspace‐Gas Chromatography: Theory and Practice. New York: John Wiley & Sons.

[mas21835-bib-0077] Kumar S , Huang JZ , Abbassi‐Ghadi N , Španěl P , Smith D , Hanna GB. Selected ion flow tube mass spectrometry analysis of exhaled breath for volatile organic compound profiling of esophago‐gastric cancer. Anal. Chem. 2013;85:6121‐6128. 10.1021/ac4010309 23659180

[mas21835-bib-0078] Kuuliala L , Jain N , De Baets B , Devlieghere F. Identifying Potential Volatile Spoilage Indicators in Shredded Carrot Using SIFT‐MS. In: Beauchamp JD, ed. Dynamic Flavor: Capturing Aroma Using Real‐Time Mass Spectrometry. American Chemical Society; 2021:107‐122. 10.1021/bk-2021-1402.ch009

[mas21835-bib-0079] Kuuliala L , Sader M , Solimeo A , Perez‐Fernandez R , Vanderroost M , De Baets B , De Meulenaer B , Ragaert P , Devlieghere F. Spoilage evaluation of raw Atlantic salmon (Salmo salar) stored under modified atmospheres by multivariate statistics and augmented ordinal regression. Int. J. Food Microbiol. 2019;303:46‐57. 10.1016/j.ijfoodmicro.2019.04.011 31136954

[mas21835-bib-0080] La Nasa J , Degano I , Modugno F , Guerrini C , Facchetti F , Turina V , Carretta A , Greco C , Ferraris E , Colombini MP , Ribechini E. Archaeology of the invisible: the scent of Kha and Merit. J. Archaeol. Sci. 2022;141:105577. 10.1016/j.jas.2022.105577

[mas21835-bib-0081] La Nasa J , Lomonaco T , Manco E , Ceccarini A , Fuoco R , Corti A , Modugno F , Castelvetro V , Degano I. Plastic breeze: volatile organic compounds (VOCs) emitted by degrading macro‐ and microplastics analyzed by selected ion flow‐tube mass spectrometry. Chemosphere 2021;270:128612. 10.1016/j.chemosphere.2020.128612 33127106

[mas21835-bib-0082] La Nasa J , Mattonai M , Modugno F , Degano I , Ribechini E. Comics' VOC‐abulary: study of the ageing of comic books in archival bags through VOCs profiling. Polym. Degrad. Stab. 2019;161:39‐49. 10.1016/j.polymdegradstab.2019.01.001

[mas21835-bib-0083] La Nasa J , Modugno F , Colombini MP , Degano I. Validation study of selected ion flow tube‐mass spectrometry (SIFT‐MS) in heritage science: characterization of natural and synthetic paint varnishes by portable mass spectrometry. J. Am. Soc. Mass Spectrom. 2019;30:2250‐2258. 10.1007/s13361-019-02305-4 31489561

[mas21835-bib-0084] La Nasa J , Nardella F , Modugno F , Colombini MP , Ribechini E , Degano I. SIFT‐ing archaeological artifacts: selected ion flow tube‐mass spectrometry as a new tool in archaeometry. Talanta 2020;207:8. 10.1016/j.talanta.2019.120323 31594618

[mas21835-bib-0085] Lacko M , Wang NJ , Sovova K , Pasztor P , Španěl P. Addition of fast gas chromatography to selected ion flow tube mass spectrometry for analysis of individual monoterpenes in mixtures. Atmos. Meas. Tech. 2019;12:4965‐4982. 10.5194/amt-12-4965-2019

[mas21835-bib-0086] Langford VS , Billiau C , McEwan MJ. Evaluation of the efficacy of SIFT‐MS for speciation of wastewater treatment plant odors in parallel with human sensory analysis. Environments 2020;7:90. 10.3390/environments7100090

[mas21835-bib-0087] Langford VS , Du Bruyn C , Padayachee D. An evaluation of selected ion flow tube mass spectrometry for rapid instrumental determination of paper type, origin and sensory attributes. Packag. Technol. Sci. 2021;34:245‐260. 10.1002/pts.2555

[mas21835-bib-0088] Langford VS , Graves I , McEwan MJ. Rapid monitoring of volatile organic compounds: a comparison between gas chromatography/mass spectrometry and selected ion flow tube mass spectrometry. Rapid Commun. Mass Spectrom. 2014;28:10‐18. 10.1002/rcm.6747 24285385

[mas21835-bib-0089] Langford VS , Gray JDC , Maclagan RGAR , Milligan DB , McEwan MJ. Real‐time measurements of nitrosamines in air. Int. J. Mass Spectrom. 2015;377:490‐495. 10.1016/j.ijms.2014.04.001

[mas21835-bib-0090] Langford VS , Gray JDC , McEwan MJ. Selected ion flow tube studies of several siloxanes. Rapid Commun. Mass Spectrom. 2013;27:700‐706. 10.1002/rcm.6496 23418149

[mas21835-bib-0091] Langford VS , McEwan MJ , Askey M , Barnes HA , Olerenshaw JG. Comprehensive instrumental odor analysis using SIFT‐MS: a case study. Environments 2018;5:43. 10.3390/environments5040043

[mas21835-bib-0092] Langford VS , Padayachee D , Bell KJ , Ma J. High‐throughput thermal desorption analysis of volatile compounds using selected ion flow tube mass spectrometry. Chromatog. Today 2021;14:30‐34.

[mas21835-bib-0093] Langford VS , Padayachee D , McEwan MJ , Barringer SA. Comprehensive odorant analysis for on‐line applications using selected ion flow tube mass spectrometry (SIFT‐MS). Flavour Fragr. J. 2019;34:393‐410. 10.1002/ffj.3516

[mas21835-bib-0094] Langford VS , Perkins MJ. Untargeted selected ion flow tube mass spectrometry headspace analysis: high‐throughput differentiation of virgin and recycled polyethylene pellets. Rapid Commun. Mass Spectrom. 2022;36:e9230. 10.1002/rcm.9230 34862682

[mas21835-bib-0095] Lee SH , Shin EJ , Zoh KD , Kang YS , Choi JW. Direct mass spectrometry with online headspace sample pretreatment for continuous water quality monitoring. Water 2020;12:1843. 10.3390/w12071843

[mas21835-bib-0096] Lee SL , O'Connor TF , Yang XC , Cruz CN , Chatterjee S , Madurawe RD , Moore CMV , Yu LX , Woodcock J. Modernizing pharmaceutical manufacturing: from batch to continuous production. J. Pharm. Innov. 2015;10:191‐199. 10.1007/s12247-015-9215-8

[mas21835-bib-0097] Lehnert AS , Behrendt T , Ruecker A , Pohnert G , Trumbore SE. SIFT‐MS optimization for atmospheric trace gas measurements at varying humidity. Atmos. Meas. Tech. 2020;13:3507‐3520. 10.5194/amt-13-3507-2020

[mas21835-bib-0098] Lehnert AS , Perreca E , Gershenzon J , Pohnert G , Trumbore SE. Simultaneous real‐time measurement of isoprene and 2‐methyl‐3‐buten‐2‐ol emissions from trees using SIFT‐MS. Front. Plant Sci. 2020;11:578204. 10.3389/fpls.2020.578204 33329639 PMC7728719

[mas21835-bib-0099] Lewis AC , Hopkins JR , Carslaw DC , Hamilton JF , Nelson BS , Stewart G , Dernie J , Passant N , Murrells T. An increasing role for solvent emissions and implications for future measurements of volatile organic compounds. Philosophical Trans. Royal Soc. A—Math. Phys. Eng. Sci. 2020;378:20190328. 10.1098/rsta.2019.0328 PMC7536026 32981432

[mas21835-bib-0100] Li KW , Chen LH , White SJ , Yu H , Wu XC , Gao X , Azzi M , Cen KF. Smog chamber study of the role of NH3 in new particle formation from photo‐oxidation of aromatic hydrocarbons. Sci. Total Environ. 2018;619:927‐937. 10.1016/j.scitotenv.2017.11.180 29734638

[mas21835-bib-0101] Li LJ , Cocker DR. Molecular structure impacts on secondary organic aerosol formation from glycol ethers. Atmos. Environ. 2018;180:206‐215. 10.1016/j.atmosenv.2017.12.025

[mas21835-bib-0102] Li LJ , Qi L , Cocker DR. Contribution of methyl group to secondary organic aerosol formation from aromatic hydrocarbon photooxidation. Atmos. Environ. 2017;151:133‐139. 10.1016/j.atmosenv.2016.11.064

[mas21835-bib-0103] Li WH , Li LJ , Chen CL , Kacarab M , Peng WH , Price D , Xu J , Cocker DR. Potential of select intermediate‐volatility organic compounds and consumer products for secondary organic aerosol and ozone formation under relevant urban conditions. Atmos. Environ. 2018;178:109‐117. 10.1016/j.atmosenv.2017.12.019

[mas21835-bib-0104] Lobaccaro P , Mandal L , Motapothula MR , Sherburne M , Martin J , Venkatesan T , Ager JW. Initial application of selected‐ion flow‐tube mass spectrometry to real‐time product detection in electrochemical CO_2_ reduction. Energy Technol. 2018;6:110‐121. 10.1002/ente.201700628

[mas21835-bib-0105] Lubes G , Goodarzi M. GC‐MS based metabolomics used for the identification of cancer volatile organic compounds as biomarkers. J. Pharm. Biomed. Anal. 2018;147:313‐322. 10.1016/j.jpba.2017.07.013 28750734

[mas21835-bib-0106] Mandal L , Yang KR , Motapothula MR , Ren D , Lobaccaro P , Patra A , Sherburne M , Batista VS , Yeo BS , Ager JW , Martin J , Venkatesan T. Investigating the role of copper oxide in electrochemical CO_2_ reduction in real time. ACS Appl. Mater. Interfaces 2018;10:8574‐8584. 10.1021/acsami.7b15418 29437377

[mas21835-bib-0107] Mansa R , Zou S. Thermogravimetric analysis of microplastics: a mini review. Environ. Adv. 2021;5:100117. 10.1016/j.envadv.2021.100117

[mas21835-bib-0108] Markar SR , Wiggins T , Antonowicz S , Chin ST , Romano A , Nikolic K , Evans B , Cunningham D , Mughal M , Lagergren J , Hanna GB. Assessment of a noninvasive exhaled breath test for the diagnosis of oesophagogastric cancer. Jama Oncol. 2018;4:970‐976. 10.1001/jamaoncol.2018.0991 29799976 PMC6145735

[mas21835-bib-0109] Michalcikova RB , Dryahina K , Španěl P. SIFT‐MS quantification of several breath biomarkers of inflammatory bowel disease, IBD: a detailed study of the ion chemistry. Int. J. Mass Spectrom. 2016;396:35‐41. 10.1016/j.ijms.2015.12.007

[mas21835-bib-0110] Michalcikova RB , Dryahina K , Španěl P. A detailed study of the ion chemistry of alkenes focusing on heptenes aimed at their SIFT‐MS quantification. Int. J. Mass Spectrom. 2018;425:16‐21. 10.1016/j.ijms.2017.12.004

[mas21835-bib-0111] Michalčíková RB , Španěl P. A selected ion flow tube study of the ion molecule association reactions of protonated (MH^+^), nitrosonated (MNO^+^) and dehydroxidated (M‐OH)^(+)^ carboxylic acids (M) with H_2_O. Int. J. Mass Spectrom. 2014;368:15‐22. 10.1016/j.ijms.2014.04.010

[mas21835-bib-0112] Milligan DB , Francis GJ , Prince BJ , McEwan MJ. Demonstration of selected ion flow tube MS detection in the parts per trillion range. Anal. Chem. 2007;79:2537‐2540. 10.1021/ac0622678 17302391

[mas21835-bib-0113] Müller M , Piel F , Gutmann R , Sulzer P , Hartungen E , Wisthaler A. A novel method for producing NH_4_ ^+^ reagent ions in the hollow cathode glow discharge ion source of PTR‐MS instruments. Int. J. Mass Spectrom. 2020;447:116254. 10.1016/j.ijms.2019.116254

[mas21835-bib-0114] Neisser M. International roadmap for devices and systems lithography roadmap. J. Micro/Nanopatterning, Mater. Metrol. 2021;20:044601.

[mas21835-bib-0115] Olivares A , Dryahina K , Španěl P , Flores M. Rapid detection of lipid oxidation in beef muscle packed under modified atmosphere by measuring volatile organic compounds using SIFT‐MS. Food Chem. 2012;135:1801‐1808. 10.1016/j.foodchem.2012.06.075 22953926

[mas21835-bib-0116] Ozcan‐Sinir G. Detection of adulteration in extra virgin olive oil by selected ion flow tube mass spectrometry (SIFT‐MS) and chemometrics. Food Control 2020;118:107433. 10.1016/j.foodcont.2020.107433

[mas21835-bib-0117] Ozcan‐Sinir G , Barringer SA. Variety differences in garlic volatile sulfur compounds, by application of selected ion flow tube mass spectrometry (SIFT‐MS) with chemometrics. Turk. J. Agric. Forestry 2020;44:408‐416. 10.3906/tar-1910-26

[mas21835-bib-0118] Padayachee D , Langford VS. SIFTing through flavor—exploring real‐time, real‐life processes using SIFT‐MS. In: Beauchamp JD, ed. Dynamic Flavor: Capturing Aroma Release using Real-Time Mass Spectrometry. Washington: American Chemical Society; 2021. 10.1021/bk-2021-1402.ch004

[mas21835-bib-0119] Peñalver R , Arroyo‐Manzanares N , López‐García I , Hernández‐Córdoba M. An overview of microplastics characterization by thermal analysis. Chemosphere 2020;242:125170. 10.1016/j.chemosphere.2019.125170 31675574

[mas21835-bib-0120] Perkins MJ , Langford VS. Application of routine analysis procedures to a direct mass spectrometry technique: selected ion flow tube mass spectrometry (SIFT‐MS). Rev. Sep. Sci. 2021a;3:e21003.

[mas21835-bib-0121] Perkins MJ , Langford VS. Standard validation protocol for selected ion flow tube mass spectrometry methods applied to direct headspace analysis of aqueous volatile organic compounds. Anal. Chem. 2021b;93:8386‐8392. 10.1021/acs.analchem.1c01310 34101412

[mas21835-bib-0122] Perkins MJ , Langford VS. Multiple headspace extraction‐selected ion flow tube mass spectrometry (MHE‐SIFT‐MS). Part 1: a protocol for method development and transfer to routine analysis. Rev. Sep. Sci. 2022;4:e22001. 10.17145/rss.22.001

[mas21835-bib-0123] Perkins MJ , Langford VS , McEwan MJ. High‐throughput analysis of volatile compounds in air, water and soil using SIFT‐MS. Curr. Trends Mass Spectrom. 2018;16:24‐29.

[mas21835-bib-0124] Prince BJ , Milligan DB , McEwan MJ. Application of selected ion flow tube mass spectrometry to real‐time atmospheric monitoring. Rapid Commun. Mass Spectrom. 2010;24:1763‐1769. 10.1002/rcm.4574 20499321

[mas21835-bib-0125] Reyrolle M , Ghislain M , Bru N , Vallverdu G , Pigot T , Desauziers V , Le Bechec M. Volatile fingerprint of food products with untargeted SIFT‐MS data coupled with mixOmics methods for profile discrimination: application case on cheese. Food Chem. 2022;369:130801. 10.1016/j.foodchem.2021.130801 34450514

[mas21835-bib-0126] Romanias MN , Zeineddine MN , Gaudion V , Lun XX , Thevenet F , Riffault V. Heterogeneous interaction of isopropanol with natural gobi dust. Environ. Sci. Technol. 2016;50:11714‐11722. 10.1021/acs.est.6b03708 27680094

[mas21835-bib-0127] Ross BM , Puukila S , Malik I , Babay S , Lecours M , Agostino A , Wondimu T , Khaper N. The Use of SIFT‐MS to investigate headspace aldehydes as markers of lipid peroxidation. Curr. Anal. Chem. 2013;9:600‐613. 10.2174/15734110113099990025

[mas21835-bib-0128] Sharma NK , Choct M , Dunlop MW , Wu SB , Castada HZ , Swick RA. Characterisation and quantification of changes in odorants from litter headspace of meat chickens fed diets varying in protein levels and additives. Poult. Sci. 2017;96:851‐860. 10.3382/ps/pew309 27664201

[mas21835-bib-0129] Sharma NK , Keerqin C , Wu SB , Choct M , Swick RA. Emissions of volatile odorous metabolites by Clostridium perfringens—in vitro study using two broth cultures. Poult. Sci. 2017;96:3291‐3297. 10.3382/ps/pex129 28651342

[mas21835-bib-0130] Shestivska V , Antonowicz SS , Dryahina K , Kubišta J , Smith D , Španěl P. Direct detection and quantification of malondialdehyde vapour in humid air using selected ion flow tube mass spectrometry supported by gas chromatography/mass spectrometry. Rapid Commun. Mass Spectrom. 2015;29:1069‐1079. 10.1002/rcm.7198 26044275

[mas21835-bib-0131] Shestivska V , Dryahina K , Nunvar J , Sovova K , Elhottova D , Nemec A , Smith D , Španěl P. Quantitative analysis of volatile metabolites released in vitro by bacteria of the genus Stenotrophomonas for identification of breath biomarkers of respiratory infection in cystic fibrosis. J. Breath Res. 2015;9:027104. 10.1088/1752-7155/9/2/027104 25830686

[mas21835-bib-0132] Shestivska V , Kolivoska V , Kubista J , Smith D , Španěl P. Selected ion flow tube mass spectrometry analyses of isobaric compounds methanol and hydrazine in humid air. Rapid Commun. Mass Spectrom. 2020;34:e8744. 10.1002/rcm.8744 32022319

[mas21835-bib-0133] Shi Z , Vu T , Kotthaus S , Harrison RM , Grimmond S , Yue S , Zhu T , Lee J , Han Y , Demuzere M , Dunmore RE , Ren L , Liu D , Wang Y , Wild O , Allan J , Acton WJ , Barlow J , Barratt B , Beddows D , Bloss WJ , Calzolai G , Carruthers D , Carslaw DC , Chan Q , Chatzidiakou L , Chen Y , Crilley L , Coe H , Dai T , Doherty R , Duan F , Fu P , Ge B , Ge M , Guan D , Hamilton JF , He K , Heal M , Heard D , Hewitt CN , Hollaway M , Hu M , Ji D , Jiang X , Jones R , Kalberer M , Kelly FJ , Kramer L , Langford B , Lin C , Lewis AC , Li J , Li W , Liu H , Liu J , Loh M , Lu K , Lucarelli F , Mann G , McFiggans G , Miller MR , Mills G , Monk P , Nemitz E , O'Connor F , Ouyang B , Palmer PI , Percival C , Popoola O , Reeves C , Rickard AR , Shao L , Shi G , Spracklen D , Stevenson D , Sun Y , Sun Z , Tao S , Tong S , Wang Q , Wang W , Wang X , Wang X , Wang Z , Wei L , Whalley L , Wu X , Wu Z , Xie P , Yang F , Zhang Q , Zhang Y , Zhang Y , Zheng M. Introduction to the special issue “In‐depth study of air pollution sources and processes within Beijing and its surrounding region (APHH‐Beijing)”. Atmos. Chem. Phys. 2019;19:7519‐7546. 10.5194/acp-19-7519-2019

[mas21835-bib-0134] Smith D , Adams NG , Miller TM. Laboratory study of reactions of N^+^, N_2_ ^+^, N_3_ ^+^, N_4_ ^+^, O^+^, O_2_ ^+^, and NO^+^ ions with several molecules at 300K. J. Chem. Phys. 1978;69:308‐318.

[mas21835-bib-0135] Smith D , Bloor R , George C , Pysanenko A , Španěl P. Release of toxic ammonia and volatile organic compounds by heated cannabis and their relation to tetrahydrocannabinol content. Anal. Methods 2015;7:4104‐4110. 10.1039/C5AY00593K

[mas21835-bib-0136] Smith D , Chippendale TWE , Dryahina K , Španěl P. SIFT‐MS analysis of nose‐exhaled breath; mouth contamination and the influence of exercise. Curr. Anal. Chem. 2013;9:565‐575.

[mas21835-bib-0137] Smith D , Chippendale TWE , Španěl P. Selected ion flow tube, SIFT, studies of the reactions of H_3_O^+^, NO^+^ and O_2_ ^+^ with some biologically active isobaric compounds in preparation for SIFT‐MS analyses. Int. J. Mass Spectrom. 2011;303:81‐89. 10.1016/j.ijms.2011.01.005

[mas21835-bib-0138] Smith D , Chippendale TWE , Španěl P. Minimising the effects of isobaric product ions in SIFT‐MS quantification of acetaldehyde, dimethyl sulphide and carbon dioxide. Curr. Anal. Chem. 2013;9:550‐557.

[mas21835-bib-0139] Smith D , Chippendale TWE , Španěl P. Reactions of the selected ion flow tube mass spectrometry reagent ions H_3_O^+^ and NO^+^ with a series of volatile aldehydes of biogenic significance. Rapid Commun. Mass Spectrom. 2014;28:1917‐1928. 10.1002/Rcm.6977 25088135

[mas21835-bib-0140] Smith D , Diskin AM , Ji YF , Španěl P. Concurrent use of H_3_O^+^, NO^+^, and O_2_ ^+^ precursor ions for the detection and quantification of diverse trace gases in the presence of air and breath by selected ion‐flow tube mass spectrometry. Int. J. Mass Spectrom. 2001;209:81‐97.

[mas21835-bib-0141] Smith D , McEwan MJ , Španěl P. Understanding gas phase ion chemistry is the key to reliable selected ion flow tube‐mass spectrometry analyses. Anal. Chem. 2020;92:12750‐12762. 10.1021/acs.analchem.0c03050 32857492

[mas21835-bib-0142] Smith D , Pysanenko A , Španěl P. Ionic diffusion and mass discrimination effects in the new generation of short flow tube SIFT‐MS instruments. Int. J. Mass Spectrom. 2009;281:15‐23. 10.1016/j.ijms.2008.11.007

[mas21835-bib-0143] Smith D , Sovova K , Dryahina K , Dousova T , Drevinek P , Španěl P. Breath concentration of acetic acid vapour is elevated in patients with cystic fibrosis. J. Breath Res. 2016a;10:6. 10.1088/1752-7155/10/2/021002 27184114

[mas21835-bib-0144] Smith D , Sovová K , Dryahina K , Doušová T , Dřevínek P , Španěl P. Breath concentration of acetic acid vapour is elevated in patients with cystic fibrosis. J. Breath Res. 2016b;10:021002. 27184114 10.1088/1752-7155/10/2/021002

[mas21835-bib-0145] Smith D , Sovová K , Španěl P. A selected ion flow tube study of the reactions of H_3_O^+^, NO^+^ and O_2_ ^+^ with seven isomers of hexanol in support of SIFT‐MS. Int. J. Mass Spectrom. 2012;319:25‐30. 10.1016/j.ijms.2012.03.009

[mas21835-bib-0146] Smith D , Španěl P. Ions in the terrestrial atmosphere and in interstellar clouds. Mass Spectrom. Rev. 1995;14:255‐278.

[mas21835-bib-0147] Smith D , Španěl P. Application of ion chemistry and the SIFT technique to the quantitative analysis of trace gases in air and on breath. Int. Rev. Phys. Chem. 1996a;15:231‐271.

[mas21835-bib-0148] Smith D , Španěl P. The novel selected‐ion flow tube approach to trace gas analysis of air and breath. Rapid Commun. Mass Spectrom. 1996b;10:1183‐1198. 8759327 10.1002/(SICI)1097-0231(19960731)10:10<1183::AID-RCM641>3.0.CO;2-3

[mas21835-bib-0149] Smith D , Španěl P. Selected ion flow tube mass spectrometry (SIFT‐MS) for on‐line trace gas analysis. Mass Spectrom. Rev. 2005;24:661‐700. 10.1002/mas.20033 15495143

[mas21835-bib-0150] Smith D , Španěl P. Ambient analysis of trace compounds in gaseous media by SIFT‐MS. Analyst 2011;136:2009‐2032. 10.1039/c1an15082k 21431189

[mas21835-bib-0151] Smith D , Španěl P. Ternary association reactions of H_3_O^+^, NO^+^ and O_2_ ^+•^ with N_2_, O_2_, CO_2_ and H_2_O; implications for selected ion flow tube mass spectrometry analyses of air and breath. Rapid Commun. Mass Spectrom. 2022;36:e9241. 10.1002/rcm.9241 34904315

[mas21835-bib-0152] Smith D , Španěl P , Davies S. Trace gases in breath of healthy volunteers when fasting and after a protein‐calorie meal: a preliminary study. J. Appl. Physiol. 1999;87:1584‐1588. 10562594 10.1152/jappl.1999.87.5.1584

[mas21835-bib-0153] Smith D , Španěl P , Dryahina K. H_3_O^+^, NO^+^ and O_2_ ^+^ reactions with saturated and unsaturated monoketones and diones; focus on hydration of product ions. Int. J. Mass Spectrom. 2019;435:173‐180. 10.1016/j.ijms.2018.10.027

[mas21835-bib-0154] Smith D , Španěl P , Enderby B , Lenney W , Turner C , Davies SJ. Isoprene levels in the exhaled breath of 200 healthy pupils within the age range 7‐18 years studied using SIFT‐MS. J. Breath Res. 2010;4:017101. 10.1088/1752-7155/4/1/017101 21386206

[mas21835-bib-0155] Smith D , Španěl P , Fryer AA , Hanna F , Ferns GAA. Can volatile compounds in exhaled breath be used to monitor control in diabetes mellitus? J. Breath Res. 2011;5:022001. 10.1088/1752-7155/5/2/022001 21512208

[mas21835-bib-0156] Smith D , Španěl P , Gilchrist FJ , Lenney W. Hydrogen cyanide, a volatile biomarker of Pseudomonas aeruginosa infection. J. Breath Res. 2013;7:044001. 10.1088/1752-7155/7/4/044001 24287489

[mas21835-bib-0157] Smith D , Španěl P , Herbig J , Beauchamp J. Mass spectrometry for real‐time quantitative breath analysis. J. Breath Res. 2014;8:027101. 10.1088/1752-7155/8/2/027101 24682047

[mas21835-bib-0158] Smith D , Španěl P , Holland TA , Al Singari W , Elder JB. Selected ion flow tube mass spectrometry of urine headspace. Rapid Commun. Mass Spectrom. 1999;13:724‐729. 10343414 10.1002/(sici)1097-0231(19990430)13:8<724::aid-rcm548>3.0.co;2-e

[mas21835-bib-0159] Smith D , Turner C , Španěl P. Volatile metabolites in the exhaled breath of healthy volunteers: their levels and distributions. J. Breath Res. 2007;1:014004. 10.1088/1752-7155/1/1/014004 21383430

[mas21835-bib-0160] Smith D , Wang TS , Pysanenko A , Španěl P. A selected ion flow tube mass spectrometry study of ammonia in mouth‐ and nose‐exhaled breath and in the oral cavity. Rapid Commun. Mass Spectrom. 2008;22:783‐789. 10.1002/rcm.3434 18275096

[mas21835-bib-0161] Smith D , Wang TS , Španěl P. A SIFT study of the reactions of H_2_ONO^+^ ions with several types of organic molecules. Int. J. Mass Spectrom. 2003;230:1‐9. 10.1016/s1387-3806(03)00341-5

[mas21835-bib-0162] Son HD , An JG , Ha SY , Kim GB , Yim UH. Development of real‐time and simultaneous quantification of volatile organic compounds in ambient with SIFT‐MS (selected ion flow tube‐mass spectrometry). J. Korean Soc. Atmos. Environ. 2018;34:393‐405. 10.5572/Kosae.2018.34.3.393

[mas21835-bib-0163] Sovová K , Dryahina K , Španěl P. Selected ion flow tube (SIFT) studies of the reactions of H_3_O^+^, NO^+^ and O_2_ ^+•^ with six volatile phytogenic esters. Int. J. Mass Spectrom. 2011;300:31‐38. 10.1016/j.ijms.2010.11.021

[mas21835-bib-0164] Sovová K , Dryahina K , Španěl P , Kyncl M , Civiš S. A study of the composition of the products of laser‐induced breakdown of hexogen, octogen, pentrite and trinitrotoluene using selected ion flow tube mass spectrometry and UV‐Vis spectrometry. Analyst 2010;135:1106‐1114. 10.1039/b926425f 20419263

[mas21835-bib-0165] Sovova K , Spesyvyi A , Bursova M , Pasztor P , Kubista J , Shestivska V , Španěl P. Time‐integrated thermal desorption for quantitative SIFT‐MS analyses of atmospheric monoterpenes. Anal. Bioanal. Chem. 2019;411:2997‐3007. 10.1007/s00216-019-01782-6 30976893

[mas21835-bib-0166] Španěl P , Dryahina K , Rejskova A , Chippendale TWE , Smith D. Breath acetone concentration; biological variability and the influence of diet. Physiol. Meas. 2011;32:N23‐N31. 10.1088/0967-3334/32/8/n01 21725144

[mas21835-bib-0167] Španěl P , Dryahina K , Smith D. A general method for the calculation of absolute trace gas concentrations in air and breath from selected ion flow tube mass spectrometry data. Int. J. Mass Spectrom. 2006;249:230‐239. 10.1016/j.ijms.2005.12.024

[mas21835-bib-0168] Španěl P , Dryahina K , Smith D. Acetone, ammonia and hydrogen cyanide in exhaled breath of several volunteers aged 4‐83 years. J. Breath Res. 2007;1:011001. 10.1088/1752-7155/1/1/011001 21383426

[mas21835-bib-0169] Španěl P , Dryahina K , Smith D. A quantitative study of the influence of inhaled compounds on their concentrations in exhaled breath. J. Breath Res. 2013;7:017106. 10.1088/1752-7155/7/1/017106 23445832

[mas21835-bib-0170] Španěl P , Dryahina K , Vicherková P , Smith D. Increase of methanol in exhaled breath quantified by SIFT‐MS following aspartame ingestion. J. Breath Res. 2015;9:047104. 10.1088/1752-7155/9/4/047104 26582819

[mas21835-bib-0171] Španěl P , Smith D. Selected ion flow tube: a technique for quantitative trace gas analysis of air and breath. Med. Biol. Eng. Comput. 1996;34:409‐419. 9039741 10.1007/BF02523843

[mas21835-bib-0172] Španěl P , Smith D. SIFT studies of the reactions of H_3_O^+^, NO^+^ and O_2_ ^+^ with a series of alcohols. Int. J. Mass Spectrom. 1997;167:375‐388.

[mas21835-bib-0173] Španěl P , Smith D. Selected ion flow tube—mass spectrometry: detection and real‐time monitoring of flavours released by food products. Rapid Commun. Mass Spectrom. 1999;13:585‐596.

[mas21835-bib-0174] Španěl P , Smith D. On‐line measurement of the absolute humidity of air, breath and liquid headspace samples by selected ion flow tube mass spectrometry. Rapid Commun. Mass Spectrom. 2001;15:563‐569. 11312505 10.1002/rcm.265

[mas21835-bib-0175] Španěl P , Smith D. Influence of weakly bound adduct ions on breath trace gas analysis by selected ion flow tube mass spectrometry (SIFT‐MS). Int. J. Mass Spectrom. 2009;280:128‐135. 10.1016/j.ijms.2008.07.021

[mas21835-bib-0176] Španěl P , Smith D. Progress in SIFT‐MS: breath analysis and other applications. Mass Spectrom. Rev. 2011;30:236‐267. 10.1002/mas.20303 20648679

[mas21835-bib-0177] Španěl P , Smith D. Advances in on‐line absolute trace gas analysis by SIFT‐MS. Curr. Anal. Chem. 2013;9:525‐539.

[mas21835-bib-0178] Španěl P , Smith D. What is the real utility of breath ammonia concentration measurements in medicine and physiology? J. Breath Res. 2018;12:027102. 10.1088/1752-7163/aa907f 28972201

[mas21835-bib-0179] Španěl P , Smith D. Dissociation of H_3_O^+^, NO^+^ and O_2_ ^+•^ reagent ions injected into nitrogen carrier gas in SIFT‐MS and reactivity of the ion fragments. Int. J. Mass Spectrom. 2020a;458:116438. 10.1016/j.ijms.2020.116438

[mas21835-bib-0180] Španěl P , Smith D. Quantification of volatile metabolites in exhaled breath by selected ion flow tube mass spectrometry, SIFT‐MS. Clin. Mass Spectrom. 2020b;16:18‐24. 10.1016/j.clinms.2020.02.001 34820516 PMC8601014

[mas21835-bib-0181] Španěl P , Sovová K , Dryahina K , Doušová T , Dřevínek P , Smith D. Acetic acid is elevated in the exhaled breath of cystic fibrosis patients. J. Cystic Fibrosis 2017;16:e17‐e18. 10.1016/j.jcf.2017.02.001 28215621

[mas21835-bib-0182] Španěl P , Spesyvyi A , Smith D. Electrostatic switching and selection of H_3_O^+^, NO^+^, and O_2_ ^+•^ reagent ions for selected ion flow‐drift tube mass spectrometric analyses of air and breath. Anal. Chem. 2019;91:5380‐5388. 10.1021/acs.analchem.9b00530 30869870

[mas21835-bib-0183] Španěl P , Swift SJ , Dryahina K , Smith D. Relative influence of helium and nitrogen carrier gases on analyte ion branching ratios in SIFT‐MS. Int. J. Mass Spectrom. 2022;476:116835. 10.1016/j.ijms.2022.116835

[mas21835-bib-0184] Španěl P , Turner C , Wang TS , Bloor R , Smith D. Generation of volatile compounds on mouth exposure to urea and sucrose: implications for exhaled breath analysis. Physiol. Meas. 2006;27:N7‐N17. 10.1088/0967-3334/27/2/n01 16400196

[mas21835-bib-0185] Španěl P , Wang TS , Smith D. Quantification of hydrogen cyanide in humid air by selected ion flow tube mass spectrometry. Rapid Commun. Mass Spectrom. 2004;18:1869‐1873. 10.1002/rcm.1566 15329882

[mas21835-bib-0186] Španěl P , Zabka J , Zymak I , Smith D. Selected ion flow tube study of the reactions of H_3_O^+^ and NO^+^ with a series of primary alcohols in the presence of water vapour in support of selected ion flow tube mass spectrometry. Rapid Commun. Mass Spectrom. 2017;31:437‐446. 10.1002/rcm.7811 27983765

[mas21835-bib-0187] Spesyvyi A , Smith D , Španěl P. Selected ion flow‐drift tube mass spectrometry: quantification of volatile compounds in air and breath. Anal. Chem. 2015;87:12151‐12160. 10.1021/acs.analchem.5b02994 26583448

[mas21835-bib-0188] Spesyvyi A , Smith D , Španěl P. Ion chemistry at elevated ion‐molecule interaction energies in a selected ion flow‐drift tube: reactions of H_3_O^+^, NO^+^ and O_2_ ^+^ with saturated aliphatic ketones. Phys. Chem. Chem. Phys. 2017;19:31714‐31723. 10.1039/c7cp05795d 29165483

[mas21835-bib-0189] Spesyvyi A , Sovova K , Španěl P. In‐tube collision‐induced dissociation for selected ion flow‐drift tube mass spectrometry, SIFDT‐MS: a case study of NO^+^ reactions with isomeric monoterpenes. Rapid Commun. Mass Spectrom. 2016;30:2009‐2016. 10.1002/rcm.7679 27459885

[mas21835-bib-0190] Spesyvyi A , Španěl P , Sovova K. Styrene radical cations for chemical ionization mass spectrometry analyses of monoterpene hydrocarbons. Rapid Commun. Mass Spectrom. 2019;33:1870‐1876. 10.1002/rcm.8556 31418494

[mas21835-bib-0191] Stefanuto PH , Zanella D , Vercammen J , Henket M , Schleich F , Louis R , Focant JF. Multimodal combination of GC x GC‐HRTOFMS and SIFT‐MS for asthma phenotyping using exhaled breath. Sci. Rep. 2020;10:16159. 10.1038/s41598-020-73408-2 32999424 PMC7528084

[mas21835-bib-0192] Su T , Chesnavich WJ. Parametrization of the ion–polar molecule collision rate constant by trajectory calculations. J. Chem. Phys. 1982;76:5183‐5185. 10.1063/1.442828

[mas21835-bib-0193] Sumonsiri N , A Barringer S. Application of SIFT‐MS in monitoring volatile compounds in fruits and vegetables. Curr. Anal. Chem. 2013;9:631‐641.

[mas21835-bib-0194] Swift SJ , Smith D , Dryahina K , Omezzine Gnioua M , Španěl P. Kinetics of reactions of NH_4_ ^+^ with some biogenic organic molecules and monoterpenes in He and N_2_ carrier gases: a potential SIFT‐MS reagent ion. Rapid Commun. Mass Spectrom. 2022;36:e9328. 10.1002/rcm.9328 35603529

[mas21835-bib-0195] Teranish R , Robinson AB , Cary P , Mon TR , Pauling L. Gas‐chromatography of volatiles from breath and urine. Anal. Chem. 1972;44:18‐20. 10.1021/ac60309a012 5006888

[mas21835-bib-0196] Turner C , Španěl P , Smith D. A longitudinal study of breath isoprene in healthy volunteers using selected ion flow tube mass spectrometry (SIFT‐MS). Physiol. Meas. 2006;27:13‐22. 10.1088/0967-3334/27/1/002 16365507

[mas21835-bib-0197] Turner C , Walton C , Hoashi S , Evans M. Breath acetone concentration decreases with blood glucose concentration in type I diabetes mellitus patients during hypoglycaemic clamps. J. Breath Res. 2009;3:046004. 10.1088/1752-7155/3/4/046004 21386197

[mas21835-bib-0198] USP . 2019a. 〈467〉 Residual Solvents. United States Pharmacopedia, the United States Pharmacopedial Convention.

[mas21835-bib-0199] USP . 2019b. 〈1467〉 Residual Solvents Verification of Compendial Procedures and Validation of Alternative Procedures. United States Pharmacopedia, the United States Pharmacopedial Convention.

[mas21835-bib-0200] Van Huffel K , Heynderickx PM , Dewulf J , Van Langenhove H. 2012. Measurement of Odorants in Livestock Buildings: SIFT‐MS and TD‐GC‐MS. In: DelRosso R, Pierucci S, Klemes JJ, Editors. Nose 2012: 3rd International Conference on Environmental Odour Monitoring and Control. p 67‐72. 10.3303/cet1230012

[mas21835-bib-0201] Vendel I , Hertog M , Nicolai B. Fast analysis of strawberry aroma using SIFT‐MS: a new technique in postharvest research. Postharvest Biol. Technol. 2019;152:127‐138. 10.1016/j.postharvbio.2019.03.007

[mas21835-bib-0202] Viggiano AA , Paulson JF. Temperature‐dependence of associative detachment reactions. J. Chem. Phys. 1983;79:2241‐2245. 10.1063/1.446073

[mas21835-bib-0203] Volckaert D , Ebude DEL , Van Langenhove H. SIFT‐MS analysis of the removal of dimethyl sulphide, n‐hexane and toluene from waste air by a two phase partitioning bioreactor. Chem. Eng. J. 2016;290:346‐352. 10.1016/j.cej.2016.01.057

[mas21835-bib-0204] Wagner RL , Farren NJ , Davison J , Young S , Hopkins JR , Lewis AC , Carslaw DC , Shaw MD. Application of a mobile laboratory using a selected‐ion flow‐tube mass spectrometer (SIFT‐MS) for characterisation of volatile organic compounds and atmospheric trace gases. Atmos. Meas. Tech. 2021;14:6083‐6100. 10.5194/amt-14-6083-2021

[mas21835-bib-0205] Walgraeve C , Bruneel J , Van Huffel K , Demeestere K , Vincze L , De Meulenaer B , Van Langenhove H. Sorption behaviour of targeted volatile organic compounds on airborne particulate matter using selected ion flow tube mass spectrometry. Biosyst. Eng. 2015;131:84‐94. 10.1016/j.biosystemseng.2015.01.007

[mas21835-bib-0206] Wang TS , Pysanenko A , Dryahina K , Španěl P , Smith D. Analysis of breath, exhaled via the mouth and nose, and the air in the oral cavity. J. Breath Res. 2008;2:037013. 10.1088/1752-7155/2/3/037013 21386174

[mas21835-bib-0207] Wang XJ , Romanias MN , Pei ZH , Rousseau A , Thevenet F. Uptake mechanism of acetic acid onto natural gobi dust. Acs Earth. Space Chem. 2020;4:1650‐1662. 10.1021/acsearthspacechem.0c00168

[mas21835-bib-0208] Wood JE , Gill BD , Longstaff WM , Crawford RA , Indyk HE , Kissling RC , Lin YH , Bergonia CA , Davis LM , Matuszek A. Dairy product quality using screening of aroma compounds by selected ion flow tube‐mass spectrometry: a chemometric approach. Int. Dairy J. 2021;121:105107. 10.1016/j.idairyj.2021.105107

[mas21835-bib-0209] Yeoman AM , Heeley‐Hill AC , Shaw M , Andrews SJ , Lewis AC. Inhalation of VOCs from facial moisturizers and the influence of dose proximity. Indoor Air 2022;32:12948. 10.1111/ina.12948 34816489

[mas21835-bib-0210] Yeoman AM , Shaw M , Carslaw N , Murrells T , Passant N , Lewis AC. Simplified speciation and atmospheric volatile organic compound emission rates from non‐aerosol personal care products. Indoor Air 2020;30:459‐472. 10.1111/ina.12652 32034823 PMC7217173

[mas21835-bib-0211] Yu JX , Castada HZ , Huang XS , Barringer SA. Comparison of encapsulation of garlic oil with ‐, ‐, and ‐cyclodextrin using selected ion flow tube‐mass spectrometry (SIFT‐MS). J. Food Processing. Preserv. 2019;43:e13865. 10.1111/jfpp.13865

[mas21835-bib-0212] Zeineddine MN , Romanias MN , Gaudion V , Riffault V , Thevenet F. Heterogeneous interaction of isoprene with natural gobi dust. Acs Earth Space Chem. 2017;1:236‐243. 10.1021/acsearthspacechem.7b00050

[mas21835-bib-0213] Zeineddine MN , Romanias MN , Riffault V , Thevenet F. Heterogeneous interaction of various natural dust samples with isopropyl alcohol as a probe VOC. J. Phys. Chem. A 2018;122:4911‐4919. 10.1021/acs.jpca.8b02034 29756775

[mas21835-bib-0214] Zhu JH , Nones C , Li Y , Milligan D , Prince B , Polster M , Dearth M. Ultra‐trace real time VOC measurements by SIFT‐MS for VIAQ. SAE Int. J. Engines 2017;10:1815‐1819. 10.4271/2017-01-0989

[mas21835-bib-0215] Zymak I , Zabka J , Polasek M , Španěl P , Smith D. A pilot study of ion‐molecule reactions at temperatures relevant to the atmosphere of titan. Orig. Life Evol. Biosph. 2016;46:533‐538. 10.1007/s11084-016-9499-9 27108425

[mas21835-bib-0216] Zymak I , Žabka J , Polášek M , Španěl P , Smith D. Experimental study of the reaction of NO_2_ ^−^ ions with CO_2_ molecules at temperatures and energies relevant to the Martian atmosphere. Icarus 2020;335:113416. 10.1016/j.icarus.2019.113416

